# Jingzhi Guanxin Oral Liquids Attenuate Atherosclerotic Coronary Heart Disease via Modulating Lipid Metabolism and PPAR-Related Targets

**DOI:** 10.3390/ph17060784

**Published:** 2024-06-14

**Authors:** Xinning Wang, Tao Hu, Yuliang Jiang, Yan He, Peibo Li, Wei Peng, Yonggang Wang, Weiwei Su

**Affiliations:** Guangdong Engineering and Technology Research Center for Quality and Efficacy Reevaluation of Post-Market Traditional Chinese Medicine, Guangdong Key Laboratory of Plant Resources, State Key Laboratory of Biocontrol, School of Life Sciences, Sun Yat-sen University, Guangzhou 510275, China

**Keywords:** Jingzhi Guanxin oral liquids, atherosclerotic coronary heart disease, network pharmacology, metabolomics, lipid metabolism, PPARs

## Abstract

Jingzhi Guanxin Oral Liquids (JZGX), a traditional Chinese medicine formulation prepared from the decoction of five herbs, has been utilized to relieve chest pain with coronary artery disease (CAD). However, the chemical composition and therapeutic mechanisms of JZGX remain obscured. In this research, the potential targets and pathways of JZGX against CAD were anticipated through network pharmacology based on analyzing its chemical constituents using UPLC-Q-TOF-MS/MS. One hundred seven ingredients in JZGX were identified. The 39 active chemicals and 37 key targets were screened, and CAD-related signaling pathways were clustered, mainly associated with lipid metabolism. Subsequently, the atherosclerotic CAD animal model employing 24 weeks of high-fat diet (HFD) ApoE^−/−^ mice was constructed to investigate the JZGX efficacy and underlying mechanisms validating network forecasts. The histological staining examination and cardiovascular biomarker tests confirmed that JZGX reduced plaque formation in the aorta and decreased blood lipids in vivo. It featured anti-inflammatory, anti-thrombotic, and myocardial protective effects. JZGX prevented excessive lipid deposits and inflammation within the liver and exhibited hepatoprotective properties. Serum untargeted metabolomics analysis indicated that JZGX ameliorated metabolic abnormalities in atherosclerotic CAD mice and prompted lipid metabolism, especially linoleic acid. The PPARs and attached critical targets (SREBP1, FASN, PTGS2, and CYP3A), filtered from the networks and connected with lipid metabolism, were dramatically modulated through JZGX administration, as revealed by western blotting. The molecular docking outcomes showed that all 39 active ingredients in JZGX had good binding activity with PPARα and PPARγ. These findings illustrate that JZGX alleviates atherosclerotic CAD progression by remodeling the lipid metabolism and regulating PPAR-related proteins.

## 1. Introduction

Coronary artery disease (CAD) is the primary cause of death globally, contributing to over 7 million death cases a year [1]. Particularly in China, 126.91/100,000 citizens and 135.88/100,000 villagers died of CAD in 2020. The fatality and prevalence rate of CAD have continued the upward trend since 2012 [2]. CAD arises from atherosclerosis in the coronary arteries, where cholesterol plaques deposit. It can induce lumen stenosis, causing myocardial ischemia or necrosis [3]. Atherosclerotic lesions are distinguished by the accumulation and conversion of lipids, smooth muscle cells, inflammatory cells, and necrotic cell fragments underneath the endothelial cells overlaid on the interior portion of the vessel wall [4]. The etiology mechanism has been attributed to the disturbed lipid metabolism and unhinged immunity leading to the chronic inflammation of vascular intima [5]. Alongside advocating a nutritionally balanced diet and active physical activity, pharmaceutical prevention and treatment strategies for CAD are necessary. The pharmacological therapy for CAD commonly used clinically includes antiplatelets, statins, calcium channel blockers, β-blockers, nitrates, ACE inhibitors, and angiotensin II antagonists [6]. Nevertheless, many of these exhibit diverse adverse effects, such as statin-associated muscle symptoms, glycuresis, neurological complaints, and gastrointestinal symptoms that are more prevalent among Chinese patients [7,8]. With millennia of clinical applications, Chinese herbal medicine has unique and notable advantages of excellent security and efficacy, which additionally play an essential role in treating CAD [9,10].

Jingzhi Guanxin Oral Liquids (JZGX) originates from the herbal prescription of Guanxin Ⅱ, created by the renowned eastern medicine physician Mr. Guo Shikui. JZGX is a Chinese patent medicine prepared by the water decoction of five medical herbs: *Salviae Miltiorrhizae Radix et Rhizoma* (Danshen), *Paeoniae Radix Rubra* (Chishao), *Chuanxiong Rhizoma* (Chuanxiong), *Carthami Flos* (Honghua), and *Dalbergiae Odoriferae Lignum* (Jiangxiang). It has been authorized by the China Food and Drug Administration, is presently recorded in the Chinese Pharmacopoeia (2020 edition), and has shown favorable therapeutic effects and safety in clinical therapy. JZGX is used to treat chest pain resulting from internal congestion, chest tightness, precordial tingling, and angina pectoris associated with coronary heart disease. From the perspective of Traditional Chinese Medicine (TCM), pain is due to Qi-blood stagnation and Qi-blood deficiency, as described in *Huangdi Neijing*. The dysfunction of Qi and blood is the primary pathogenesis of CAD and further forms different Qi-blood syndromes. Danshen (monarch herb), Chishao (ministerial herb), and Honghua can eliminate blood stasis, while Chuanxiong and Jiangxiang stimulate and regulate Qi. The synergy of five herbs promotes blood circulation, unblocks the meridians, and alleviates pain, thus curing CAD. Meanwhile, contemporary pharmacological research demonstrates that five herbs possess variable degrees of anti-thrombotic, anti-inflammatory, antioxidant, analgesic, and other cardiovascular protective actions [11,12,13,14,15].

The intricate chemical component of Chinese herbal medicine and its synergistic effects present challenges in clarifying the molecular mechanism. Network pharmacology, based on system biology and bioinformatics, can be used to analyze the impact of multi-component drugs at the system level. It can illuminate the mechanism of herbal medicine through network construction about “drug-ingredient-target-disease” [16]. Metabolomics has been exploited to find potential biomarkers for pharmacological intervention with a high-throughput and comprehensive approach. The integration of network pharmacology and metabolomics provides an intensive approach that covers the function of TCM formulation [17].

Here, ultra-high-performance liquid chromatography with quadrupole time-of-flight mass spectrometry (UPLC-Q-TOF-MS/MS) was employed to determine the components in JZGX. The network pharmacology was subsequently applied to forecast the efficacious chemicals, possible target genes, and attached pathways of JZGX opposing CAD. A 24-week high-fat diet (HFD) ApoE^−/−^ mice model of atherosclerotic CAD was established. The therapeutic efficacy was assessed by a series of in vivo experiments, including histological staining measurements of the aortic tree, root, and liver, as well as cardiovascular indicator tests in the serum. The untargeted serum metabolomics technique, western blotting, and molecular docking verification were used to investigate its mechanisms thoroughly. 

## 2. Results

### 2.1. Chemical Ingredients of JZGX

**Figure 1:**
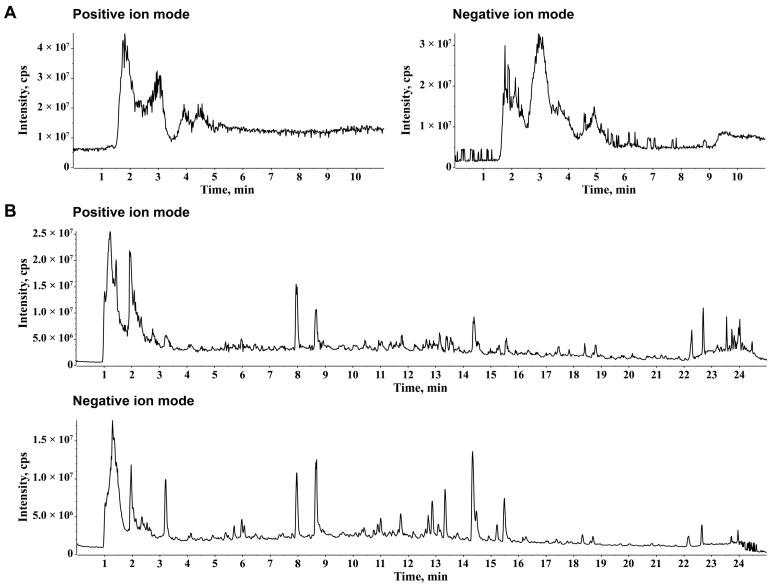
Identification of JZGX chemical composition. The total ion chromatograms in (**A**) hydrophilic interaction liquid chromatography with mass spectrometry (HILIC-MS) and (**B**) reversed phase liquid chromatography with mass spectrometry (RPLC-MS).

One hundred seven chemical components of JZGX were identified, including 42 flavonoids, 22 phenylpropanoids, eight terpenoids, eight alkaloids, seven saccharides, seven organic acids, six phenolic acids, and six phthalides. The total ion chromatograms of JZGX are shown in Figure 1. Table S1 presents the MS fragment ions of 107 compounds detected in both negative and positive ion modes, their retention times, and precise molecular weights. The constituents of JZGX were utilized for subsequent network pharmacology analysis.

### 2.2. Key Targets for JZGX in the Treatment of CAD

**Figure 2:**
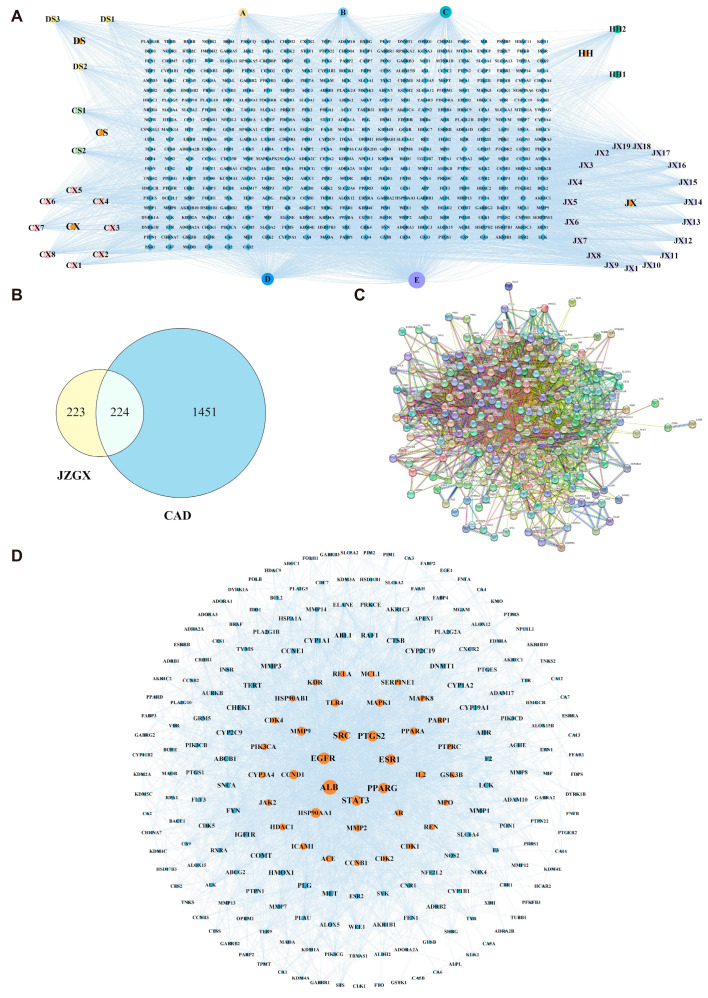
Network analysis of JZGX anti-CAD targets. (**A**) The interaction network between JZGX active components and corresponding targets. (**B**) Venn diagram of the targets associated with JZGX active components and CAD-related targets. (**C**) The PPI network of intersection targets. (**D**) Optimized network of 224 JZGX anti-CAD targets (37 key targets are shown in orange nodes).

The 39 active ingredients in JZGX were screened through the SwissADME Database (Table S2). A total of 447 potential targets, corresponding to 39 components of JZGX, were obtained from the SwissTargetPrediction Database. The network between 39 active chemical components and 447 targets was visualized (Figure 2A). Additionally, 1675 CAD targets were acquired from the Drugbank, OMIM, DisGeNET, and CTD databases. The 224 targets of JZGX anti-CAD were collected through the intersections of the targets related to JZGX components and CAD targets (Figure 2B). The protein-protein interactions (PPI) network, which comprised 224 nodes and 4642 edges, was constructed using the STRING Database (Figure 2C). Overall, 37 nodes with higher values (“Degree” > 68, “Betweenness centrality” > 0.0018, “Closeness centrality” > 0.4467) were filtered as the critical targets of JZGX in treating CAD (Figure 2D, Table S3).

### 2.3. Enrichment Analysis and “Components-Targets-Pathways” Network Construction

**Figure 3:**
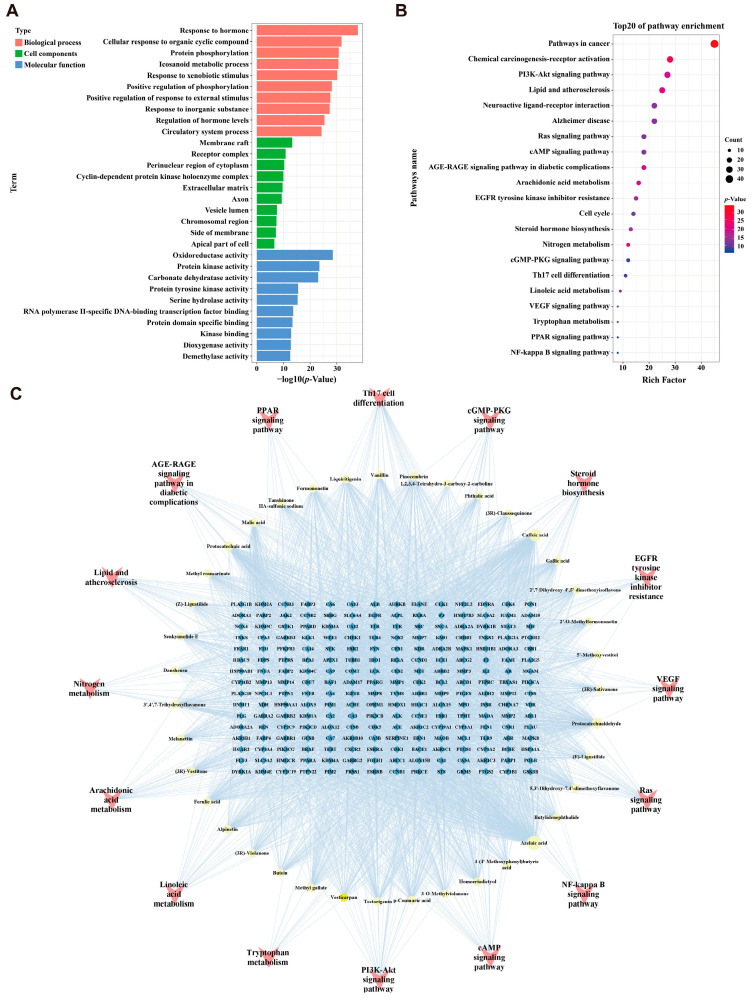
Mechanisms for the prediction of JZGX in the treatment of CAD. (**A**) GO analysis (the top 10 terms of biological processes, cell components, and molecular function enrichment). (**B**) The top 20 pathways of KEGG enrichment analysis. (**C**) The components-targets-pathways network of JZGX.

Enrichment using Gene Ontology (GO) and the Kyoto Encyclopedia of Genes and Genomes (KEGG) was conducted for the 224 JZGX anti-CAD targets. The top 10 enriched GO terms revealed that JZGX played a curative role in CAD by modulating biological processes such as the circulatory system process, extracellular matrix, and oxidoreductase activity (Figure 3A). The top 20 pathways of KEGG enrichment are displayed in Figure 3B. The network of JZGX components, targets, and pathways was established based on 39 active chemical components, 224 potential targets, and 15 KEGG pathways strongly related to CAD (Figure 3C). The enriched pathways were interacted with by the multiple common targets, indicating that JZGX might have a therapeutic effect through several signaling pathways. Among them, many signaling pathways of JZGX intervention in CAD are relative to lipid metabolism, including lipid and atherosclerosis, steroid hormone biosynthesis, linoleic acid metabolism, and peroxisome proliferator-activated receptor (PPAR) signaling pathway. Thus, a high-fat diet ApoE^−/−^ mice atherosclerotic CAD model was further conducted to investigate the effectiveness of JZGX and to ascertain the biological mechanism predicted from network pharmacology.

### 2.4. JZGX Inhibited the HFD-Induced Formation of Atherosclerotic Plaques

**Figure 4:**
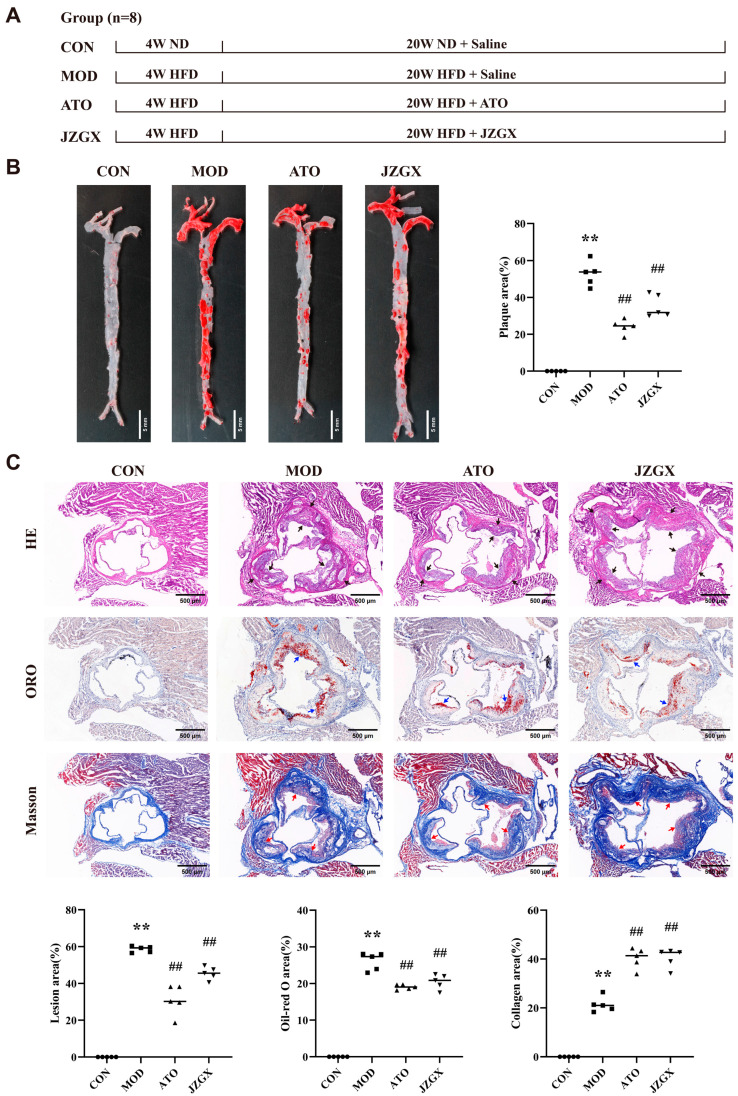
JZGX prevented the aortic plaque development in ApoE^−/−^ mice. (**A**) Animal verification experiments on the design of JZGX therapy for atherosclerotic plaque production. (**B**) The Oil Red O staining of the arterial tree and the plaque area were quantitatively analyzed. (**C**) The aortic root was subjected to HE, Oil Red O, and Masson staining techniques, resulting in quantifying the lesion area, Oil Red O region, and collagen area. Black arrows indicate the area of the atherosclerotic lesion. Blue arrows point to the lipid deposition area. Red arrows mark the collagen deposition area. The data are presented as means ± SD (*n* = 5). ** *p* < 0.01 vs. CON group, ^##^ *p* < 0.01 vs. MOD group.

A 24-week high-fat diet on ApoE^−/−^ mice was administered to induce an atherosclerotic CAD model (Figure 4A). Atherosclerosis primarily occurs in the inner walls of several middle-sized and large arteries, particularly at sites of arterial bifurcation [5]. The Oil Red O staining results about the arterial tree and cross-section of the aortic root indicated a substantial accumulation of lipids within the arterial lumina in mice subjected to the high-fat diet intake. However, interventions including atorvastatin and JZGX effectively alleviated the primary lipid accumulation in the aorta (Figure 4B,C). The Hematoxylin and Eosin (HE) staining of the aortic root showed the absence of lipid streaks and fibrous plaques in the control (CON) group of mice. The aortic root in the model (MOD) group mice had a considerable presence of lipid-filled foam cells and cholesterol crystal cracks along the blood channel wall (*p* < 0.01). In comparison to the MOD group, mice treated with atorvastatin (ATO) and JZGX exhibited a considerable reduction in the atherosclerotic plaque area of the aortic root (*p* < 0.01). The observation suggested that the administration of JZGX mitigated the development of atherosclerotic plaques (Figure 4C). The Masson staining analysis of aortic root sections displayed a high elevation in collagen content of aortic root plaques in the ATO and JZGX group mice, which contrasted with the MOD group. These findings demonstrated that JZGX treatment might benefit the stability of aortic root plaques (Figure 4C).

### 2.5. Effects of JZGX Treatments on Cardiovascular Biomarkers

**Figure 5:**
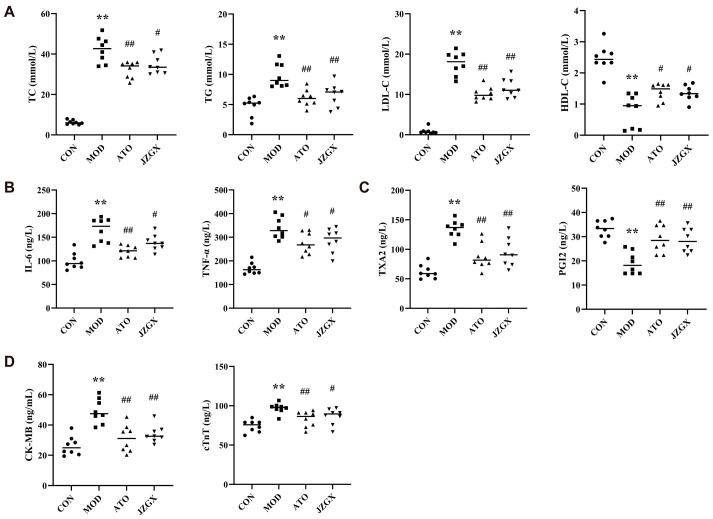
The regulation of JZGX intervention on cardiovascular mediators in ApoE^−/−^ mice. The serum quantitative tests were about (**A**) lipid profiles (TC, TG, LDL-C, and HDL-C), (**B**) inflammatory cytokines (IL-6 and TNF-α), (**C**) vascular endothelium regulators (TXA2 and PGI2), and (**D**) myocardial function indicators (CK-MB and cTnT). Data are presented as means ± SD (*n* = 8). ** *p* < 0.01 vs. CON group, ^#^ *p* < 0.05 vs. MOD group, ^##^ *p* < 0.01 vs. MOD group.

Dyslipidemia, characterized by elevated total cholesterol (TC), low-density lipoprotein cholesterol (LDL-C), triglyceride (TG), and decreased high-density lipoprotein cholesterol (HDL-C) in serum, represents a notable risk factor for cardiovascular diseases [18]. The serum in MOD group mice exhibited heightened levels of TC, TG, and LDL-C and reduced levels of HDL-C compared to the CON group, reflecting the presence of dyslipidemia (Figure 5A). JZGX treatment lessened the upregulation of TC, TG, and LDL-C, as well as raised HDL-C levels (*p* < 0.05), indicating that JZGX could affect these four serum lipid profiles and mitigate the imbalance of lipids.

Interleukin-6 (IL-6) and tumor necrosis factor-alpha (TNF-α) are essential pro-inflammatory cytokines leading to atherosclerosis formation [19,20]. The serum IL-6 and TNF-α levels were increased in the MOD group (*p* < 0.01), reflecting that the chronic administration of HFD-induced inflammatory responses in mice (Figure 5B). In the JZGX group, lowered serum levels of IL-6 and TNF-α were detected compared to the MOD group (*p* < 0.05), demonstrating that JZGX had excellent anti-inflammatory effects in the pathogenesis of CAD.

A balance between the formation and release of prostacyclin (PGI2) and thromboxane A2 (TXA2) in circulation is paramount for controlling intra-arterial thrombi formation [21]. The outcomes found that the model group mice exhibited greater serum TXA2 levels and lesser PGI2 levels than the CON group (*p* < 0.01), implying the presence of endothelial dysfunction in the MOD group. In contrast to the MOD group, JZGX intervention could substantially minimize serum TXA2 levels and augment PGI2 levels (*p* < 0.01, Figure 5C). The data revealed that JZGX exhibited anti-thrombotic properties and protected the vascular endothelium.

Creatine kinase isoenzymes (CK-MB) and cardiac troponin T (cTnT) have exceptional specificity for myocardial tissue. They are released in response to myocardial damage, making them valuable indicators of myocardial injury [22]. As shown in Figure 5D, the serum CK-MB and cTnT levels were markedly heightened in the MOD group (*p* < 0.01), reflecting that the mice in the MOD group suffered myocardial damage. In the JZGX group, mice serum displayed diminished levels of CK-MB and cTnT, signifying that JZGX had favorable effects on myocardial protection (*p* < 0.05).

### 2.6. JZGX Alleviated HFD-Induced Hepatic Lipid Accumulation

**Figure 6:**
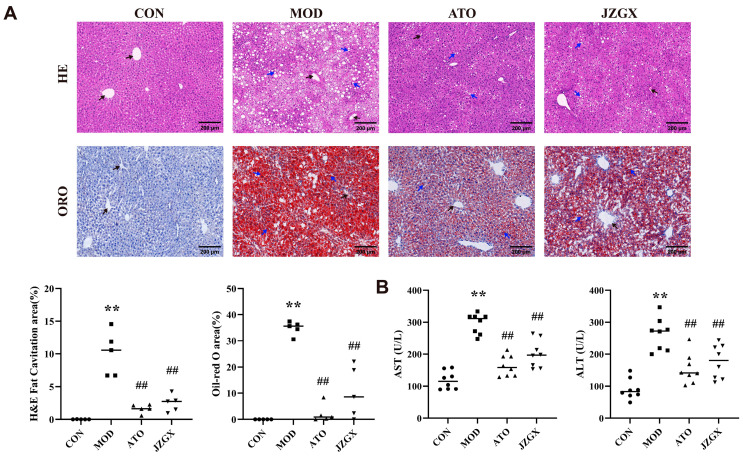
JZGX reduced liver lipid deposition in ApoE^−/−^ mice. (**A**) The HE and Oil Red O staining of the liver in mice. The fat cavitation area and the Oil Red O area were quantitatively analyzed. The data are presented as means ± SD (*n* = 5). (**B**) The detection of AST and ALT concentration in the mice serum. The data are presented as means ± SD (*n* = 8). Black arrows represent the central vein. Blue arrows denote the lipid droplets. ** *p* < 0.01 vs. CON group, ^##^ *p* < 0.01 vs. MOD group.

The liver serves as a central hub for lipid circulation [23]. Abnormal buildup of lipids in the liver is linked to an elevated CAD risk [24]. In the MOD group mice, the HE staining of the liver showed a considerable presence of hepatocyte edema and cytoplasmic vacuolation, suggesting moderate steatosis and mild infiltration of inflammatory cells. The Oil Red O staining revealed a substantial accumulation of lipids within the livers of the MOD group mice. In contrast, the HE staining of the livers in the CON group mice found no liver pathology characteristics, and the Oil Red O staining demonstrated no lipid deposition in the livers (Figure 6A). The results indicated that prolonged high-fat diet consumption led to aberrant lipid metabolism, causing lipid aggregation and pathological alterations in the liver. Compared to the MOD group, the HE staining of the liver in the ATO and JZGX group mice exhibited lower hepatocyte edema and intracellular vacuolation and reduced inflammatory cell infiltration (*p* < 0.01). The lipids of livers in the ATO and JZGX group mice were significantly descended according to the outcomes of the Oil Red O staining. These data implied that JZGX treatment suppressed the overabundance of lipids and inflammation buildup in the liver.

Hepatocytes contain large quantities of aspartate aminotransferase (AST) and alanine aminotransferase (ALT). Once hepatocytes are damaged, these enzymes get released into the bloodstream. Rising levels of AST and ALT in the serum indicate liver cell damage and implicate the risk of CAD [25,26]. As observed in Figure 6B, serum AST and ALT levels were incredibly enhanced in the MOD group mice as opposed to the CON group (*p* < 0.01), implying that cell injury and dysfunction appeared in the liver of MOD group mice. The drug intervention culminated in a notable drop of AST and ALT levels in the serum, confirming the JZGX hepatoprotective activity.

### 2.7. Serum Metabolomic Biomarkers for the JZGX Administration on CAD

**Figure 7:**
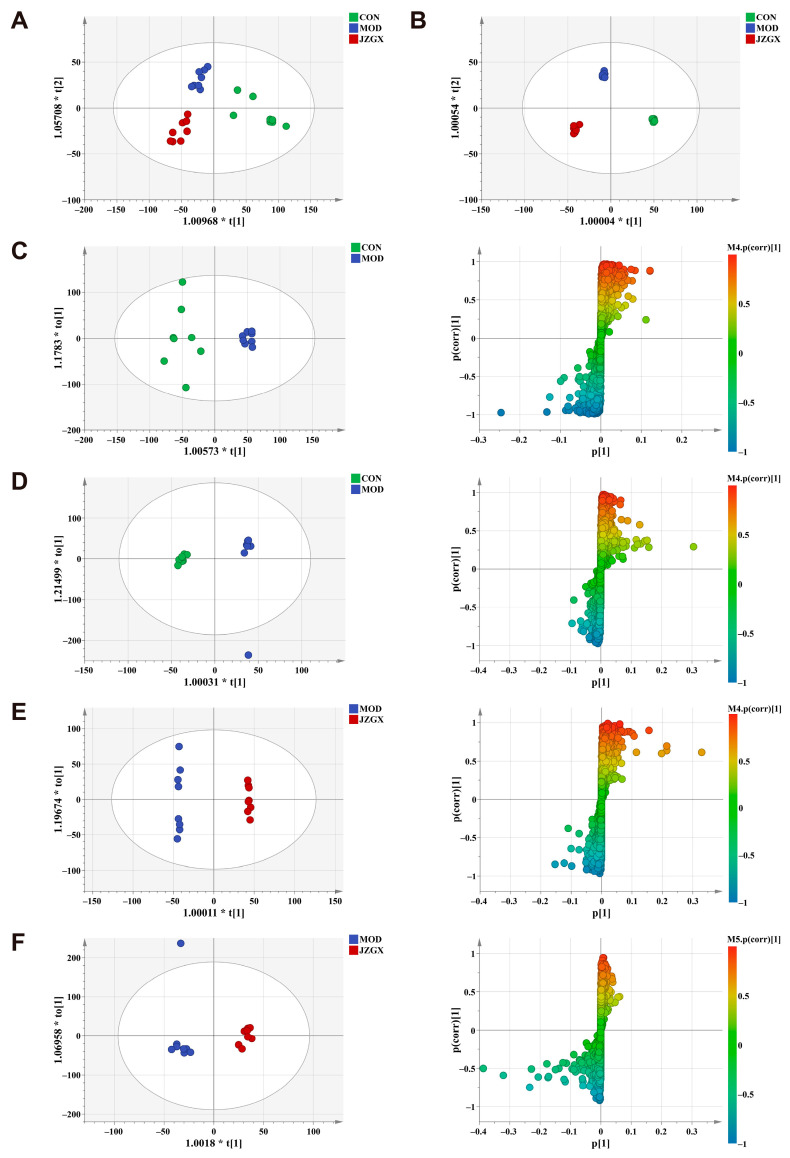
Multivariate evaluation of detected peaks in the serum metabolome. The OPLS-DA score plots in the negative (**A**) and positive (**B**) modes were collected from the CON, MOD, and JZGX groups. The OPLS-DA score plot and S-plot in the negative (**C**) and positive (**D**) mode were acquired from the CON and MOD groups. The OPLS-DA score plot and S-plot in the negative (**E**) and positive (F) mode were obtained from the MOD and JZGX groups.

During the OPLS-DA analysis of CON, MOD, and JZGX groups, the score plots revealed distinctive differentiation among the three groups in both negative and positive (Figure 7A,B) modes, which hinted that JZGX altered the metabolic profiles in mice serum. In both negative and positive ionization modes, OPLS-DA analysis was performed on the mass spectrum peak information for both the CON and MOD groups, as well as between the MOD and JZGX groups, to screen out differential metabolites. The OPLS-DA models were robust throughout a seven-round cross-validation and 200-time permutation. In the negative mode, the R2 and Q2 values from the OPLS-DA models of the CON and MOD groups were 0.719 and −0.529, while in the positive mode, they were 0.865 and −0.482 (Figure S1A,B). Comparing the MOD group with the JZGX group, the R2 and Q2 values in the OPLS-DA model were 0.994 and −0.13 in the negative mode and 0.833 and −0.285 in the positive mode (Figure S1C,D). All R2 and Q2 values on the left were lower than the original points to the right. The *p* values were all less than 0.05 in the OPLS-DA models, determined by analysis of variance (ANOVA) in the cross-validated residuals of a Y-variable [27]. The outcomes proved the validity and reliability of the OPLS-DA models without overfitting.

**Figure 8:**
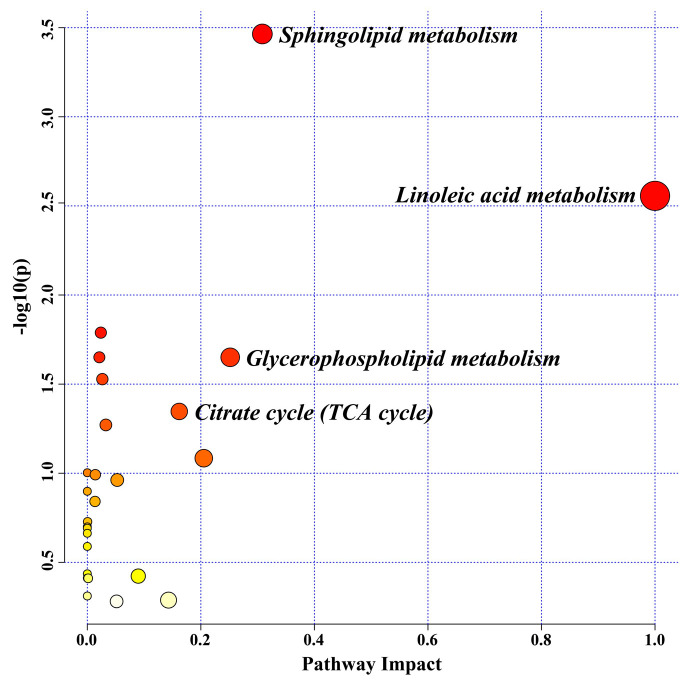
Enrichment analysis of differential metabolite pathways significantly regulated by JZGX.

The following two criteria were used to pick out differential metabolites: VIP > 1 and *p* < 0.05. Chemically identifying the feature metabolites using local and open-source databases based on mass fragmentation information and isotope peak ratios. One hundred seven possible differential biomarkers were identified, presenting a substantial correlation with the outcomes of JZGX treatment (Table S4). Serum biomarkers substantially regulated by the JZGX intervention underwent metabolic pathway enrichment. The *p* < 0.05 and impact values > 0.1 results were identified as significant metabolic pathways. The impact values arranged in descending order were linoleic acid, sphingolipid, glycerophospholipid metabolism, and citrate cycle (TCA cycle, Figure 8).

**Figure 9:**
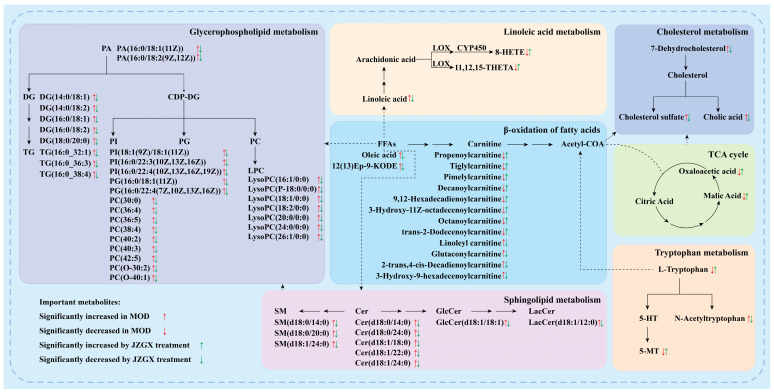
Proposed metabolic networks of JZGX on atherosclerotic CAD. Red arrows indicate differential metabolites that were significantly up- or down-regulated in the serum of the MOD group mice compared to the CON group. Green arrows represent metabolites above that were substantially altered in the serum of the JZGX group mice in contrast to the MOD group. DG, diacylglycerol; PA, phosphatidic acid; PI, phosphatidylinositol; CDP-DG, cytidine diphosphate diacylglycerol; PG, phosphatidylglycerol; PC, phosphatidylcholine; LPC, lysophosphatidylcholine; SM, sphingomyelin; Cer, ceramide; GlcCer, glucosylceramide; LacCer, lactosylceramide; FFAs, free fatty acids; LOX, lipoxygenase; CYP450, cytochrome P450 enzymes; 8-HETE, 8-hydroxyeicosatetraenoic acid; 11,12,15-THETA, 11,12,15-trihydroxyeicosatrienoic acid; 12(13)Ep-9-KODE, trans-12,13-epoxy-11-oxo-trans-9-octadecenoic acid; 5-HT, 5-hydroxytryptamine; 5-MT, 5-methoxytryptamine.

The significant differential metabolites on the above pathways were deeply analyzed. Comparatively to the CON group, many lipids of the serum in the MOD group mice expressed a substantial rise, including linoleic acid, sphingolipids, and glycerophospholipids (Figure 9). JZGX markedly decreased the levels of these lipids. Meanwhile, JZGX reversed the metabolic abnormalities of several carnitines in the β-oxidation of fatty acids, oxaloacetic acid, and malic acid in the TCA cycle, as well as metabolites in the cholesterol and tryptophan metabolism. The results stated that JZGX affected the metabolites associated with lipid metabolism to address atherosclerotic CAD.

### 2.8. JZGX Influenced the Expression of PPAR-Related Lipid Metabolism Proteins

**Figure 10:**
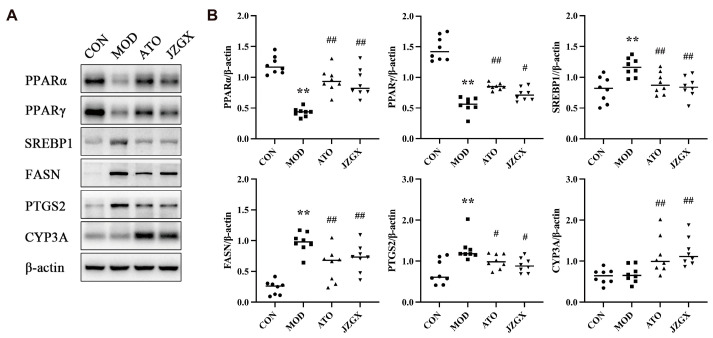
JZGX affected the expression of PPAR-related proteins in ApoE^−/−^ mice liver. (**A**) The western blotting results of PPARα, PPARγ, SREBP1, FASN, PTGS2, and CYP3A. (**B**) Quantification of grey levels as shown on western blot of these proteins. Data are presented as means ± SD (*n* = 8). ** *p* < 0.01 vs. CON group, ^#^ *p* < 0.05 vs. MOD group, ^##^ *p* < 0.01 vs. MOD group.

Activated PPARs have been proven to alleviate the whole atherogenic plasma lipid profile and suppress the inflammatory response [28]. PPARs control the various gene expressions engaged in lipid metabolic homeostasis, including sterol regulatory element-binding proteins (SREBPs), fatty acid synthase (FASN), prostaglandin-endoperoxide synthase 2 (PTGS2), and cytochrome P4503A (CYP3A) [29]. In the light of network pharmacology and serum metabolomics pathway enrichment analysis, the key PPAR-regulated lipid metabolism-linked proteins were detected. As shown in Figure 10, JZGX intervention tremendously upregulated the expression of PPARα, PPARγ, and CYP3A and downgraded the levels of SREBP1, FASN, and PTGS2 (*p* < 0.05) in the liver of the MOD mice, which implied that the JZGX could mitigate CAD by decreasing the deposition in the circulation by inhibiting lipid synthesis in the liver. According to the aforementioned target verification results, the 39 active components selected by network pharmacology were processed separately on molecular docking with PPARα and PPARγ. The docking outcomes showed that affinity < −5.0 kcal/mol between ligands and targets implied good binding activity (Table S5).

## 3. Discussion

JZGX is a compound prepared from the water decoction of TCM, in which only danshensu, salvianolic acid B, and paeoniflorin have been reported. This study uncovered the 107 chemical constituents in JZGX for the first time by combining HILIC and RPLC with TOF-MS/MS. The bioinformatic analysis of network pharmacology was applied to explore active ingredients, key targets, and potential pathways in JZGX’s treatment of CAD, which directed attention to lipid metabolism.

Coronary atherosclerosis is a lipid-driven inflammatory disease [30]. Oxidized LDL (ox-LDL) stimulates endothelial cell dysfunction. The monocytes attached to the activated endothelium differentiate into proinflammatory macrophages, continuously increasing the uptake of ox-LDL and turning into foam cells loaded with cholesterol [31]. Yellow foam cells gradually condense on the artery wall and evolve into atherosclerotic plaques. JZGX could effectively minimize plaque accumulation and stabilize it in the aorta of HFD-induced atherosclerotic CAD model mice. It lowered serum lipids and inflammatory factor levels, displaying anti-thrombotic and cardioprotective effects. The hepatic lipid homeostasis, such as the uptake, storage, re-arrangement, outflow, and synthesis of lipids, is intimately connected with CAD pathologic conditions [32]. JZGX mitigated hepatic lipid buildup, alleviated liver inflammation, and had hepatoprotective properties. Employing the serum non-targeted metabolome to search for biomarkers of JZGX treating CAD, it was found that JZGX can partly rectify the metabolic abnormalities in atherosclerotic CAD model mice. The pathway enrichment analysis of significant serum differential metabolites indicated that JZGX’s interventions for atherosclerotic CAD were associated with lipid metabolism, aligning with the network pharmacology pathway prediction.

Fatty acids are involved in CAD in manifold ways. Several cohort studies reported a notable correlation between serum total nonesterified free fatty acid concentrations and CAD mortality [33,34]. Linoleic acid, a polyunsaturated omega-6 fatty acid, activates vascular endothelial cells and disrupts endothelial integrity [35]. Linoleic acid, the predominant oxidized fatty acid found in LDL, initiates the oxidation of LDL particles [36,37]. Following the highly susceptible oxidation of linoleic acid in LDL, aldehydes and ketones form covalent bonds with apolipoprotein B, creating LDL particles no longer acknowledged by the hepatic LDL receptors [38]. The macrophage scavenger receptors recognize these modified LDL particles, forming foam cells and atherosclerosis [39]. Linoleic acid acts as a precursor, metabolized by lipoxygenases and CYP450 enzymes into mid-chain HETE, typified by 8-HETE [40]. 8-HETE exhibits intense activation of PPARα and displays limited activation of PPARγ [41]. As sensors for fatty acids and their derivatives, PPARs regulate energy homeostasis [42]. Compared to the CON group, the serum metabolome investigation revealed that the linoleic acid content in the MOD group mice was markedly elevated, while the 8-HETE content was dramatically reduced. JZGX could significantly reverse these metabolic abnormalities, spelling out JZGX’s anti-CAD effects through modulating fatty acid metabolism, Linoleic acid metabolism, and the PPAR signaling pathway. In contrast with the MOD group, 11,12,15-THETA levels in the mice serum of the JZGX group exhibited an enormous hike, and oleic acid and 12(13)Ep-9-KODE showed an immense decrease. 11,12,15-THETA, a metabolite of the 15-lipoxygenase pathway of arachidonic acid, facilitates the acetylcholine-induced vaso-relaxation via driving the K^+^ channels to hyperpolarize the smooth muscle membrane and promote relaxation [43]. Oleic acid, an omega-9 monounsaturated fatty acid, contributes to the progression of atherosclerosis by engaging in various signaling pathways that stimulate the proliferation, migration, and apoptosis of vascular smooth muscle cells [44,45]. As previously stated, the pharmacodynamic mechanism of JZGX is indivisible from the regulation of fatty acids.

Fatty acids act as the main energy source for the heart, and their β-oxidation contributes to approximately 50–70% of the heart’s ATP generation [46]. The primary function of carnitine involves transporting fatty acids across membranes and activating them during fatty acid β-oxidation in the mitochondria and peroxisomes [47]. Carnitine is a metabolic marker whose circulating level reflects the severity of CAD to a certain extent. In this study, a decrease in the levels of short-chain (propenoylcarnitine and tiglylcarnitine), medium-chain (pimelylcarnitine and decanoylcarnitine), and long-chain (3-hydroxy-11Z-octadecenoylcarnitine and 9,12-hexadecadienoylcarnitine) acylcarnitines, as well as octanoylcarnitine and isovalerylcarnitine, was observed in the serum of mice in the MOD group compared to the CON group. Conversely, the levels of glutaconylcarnitine, 2-trans,4-cis-Decadienoylcarnitine, linoleyl carnitine, and 3-Hydroxy-9-hexadecenoylcarnitine were elevated. Previous research investigations have demonstrated a decrease in the levels of decanoylcarnitine, along with a rise in linoleylcarnitine and glutarylcarnitine in the CAD patient’s plasma [48]. The substantial counteraction of metabolic imbalances by JZGX intervention suggested its potential to promote fatty acid β-oxidation and ameliorate anomalies in CAD lipid metabolism. The TCA cycle begins by combining oxaloacetate with the two-carbon acetyl-CoA generated from fatty acid β-oxidation, which releases large amounts of energy. Unlike the model group, elevated amounts of oxaloacetate and its TCA cycle metabolite, malic acid, were observed in the serum of JZGX group mice. The observations prompted the conclusion that the administration of JZGX could accelerate the mitochondrial TCA cycle to enhance carbon flux.

Lipids in the blood contain triglycerides and cholesterol, which are transferred together by triglyceride-rich lipoproteins, including very low-density lipoproteins, chylomicrons, and their cholesterol-enriched remnants. TGs are the predominant means by which fatty acids are transported and stored within cells and the plasma [49]. High TG and its glycerophospholipid metabolite levels are markers for atherogenic lipoproteins [50]. In addition, sphingolipids have shown profound biological impacts on the occurrence of atherosclerosis. Ox-LDL stimulates the synthesis of Cers via activating sphingomyelinases. In human atherosclerotic plaques, GlcCer and LacCer derived from Cer have been discovered [51]. JZGX significantly reduced the total TGs in the serum compared with the MOD group mice. The metabolic profile analyses also revealed a considerable rise in the levels of various serum lipid compounds, such as glycerophospholipids (PA, PG, PI, DG, PC, LPC, and TG), sphingolipids (SM, Cer, GlcCer, and LacCer), and cholesteryl esters (CE) in the model group mice versus the control group. The administration of JZGX effectively reversed these lipid metabolite variations, revealing that JZGX has benefits in rectifying dyslipidemia in CAD.

Excluding the metabolites mentioned above, other differential metabolites connected with cholesterol metabolism (7-dehydrocholesterol, cholesterol sulfate, and cholic acid) and tryptophan metabolism (L-tryptophan, 5-MT, and N-acetyltryptophan) were also modulated by JZGX treatment. Cholesterol sulfate is found in atherosclerotic lesions of the human aorta, which promotes platelet adhesion and may be one of the factors determining the prothrombotic potential of lesions [52]. Cholic acid plays a crucial role in intestinal cholesterol absorption. Extended administration of cholic acid induces hypercholesterolemia and the development of cholesterol gallstones in mice [53]. The content of 7-dehydrocholesterol, 5-MT, and N-acetyltryptophan in the serum of JZGX group mice was reduced compared with the MOD group, signifying that JZGX could influence blood cholesterol by blocking its synthesis.

Further, the molecular mechanism behind the therapeutic effects of JZGX on CAD through lipid metabolism was initially explored. Based on the 37 core targets screened by the PPI topological network, the enriched pathways in network pharmacology forecasts, and the influence of JZGX on lipids in efficacy research, PPAR-related lipid metabolism proteins were focused on in this research. Inhibition of PPARα enhances FASN levels, which mediates the first step in fatty acid synthesis [54]. PPARγ primarily facilitates the cellular uptake of lipids via anabolic pathways, and its activation gives rise to the upregulation of genes that mediate fatty acid uptake and trapping [55,56,57,58]. However, treatment with high-affinity agonist ligands for PPARγ, such as thiazolidinedione antidiabetic drugs, proved vasoprotective and reduced atherosclerosis in mouse models [59]. Given the fact that activation of PPARγ can induce the expression of CD36, which is associated with a consequence HDL particle production, the anti-atherosclerosis effect of PPARγ might be connected with the activation of lipid efflux mechanisms, especially reverse cholesterol transport, which assists in reducing the buildup of atherosclerotic lipids [60]. Lipid homeostasis is inseparable from the modulation of SREBPs. SREBPs govern the metabolism of cholesterol, fatty acids, triglycerides, and phospholipids by activating more than 30 genes concerning the synthesizing and ingestion of lipids. [61]. SREBP1 has been firmly stated as a crucial transcription factor that controls FASN activity in liver cells by binding the promoters [62,63,64]. Polyunsaturated fatty acids bind and mobilize PPARα to accelerate fatty acid oxidation while suppressing the activation of SREBP1 through diverse mechanisms [65]. The *CYP3A* genes encode monooxygenases, which catalyze various processes involved in drug metabolism and create cholesterol, steroids, and other lipids [66]. Interfering with the mouse CYP3A strengthens the liver cholesterol and bile acid synthesis [67]. CYP3A4 protein expression was notably reduced in liver donors with steatohepatitis [68]. Lipid peroxidation products can foster inflammatory recruitment by activating PTGS2 [69]. PTGS2 catalyzes the synthesis of prostanoids and thromboxanes from arachidonic acid, which are highly pro-inflammatory [70]. The expression of PTGS2 in the liver is elevated in cases of experimental alcoholic steatohepatitis [71]. Numerous studies have indicated that PPARs control the transcription of the lipid metabolism genes described before, impacting the synthesis and metabolism of fatty acids. Therefore, the expression of these proteins was detected, and the results revealed that JZGX regulated the PPARs and relevant lipid metabolism effectors, including SREBP1, FASN, CYP3A, and PTGS2. Activation of PPARs remodeled the lipid metabolism via fat acid beta-oxidation and lipid-genesis.

This study presented a preliminary study on the chemical composition and pharmacodynamic effects of JZGX, but it had certain limitations. For instance, there was no quantitative analysis of the chemical components in JZGX and no investigation into the in vivo metabolism of these ingredients, which would provide a clearer understanding of how JZGX functioned in treating atherosclerotic CAD. These issues will be addressed in subsequent research.

## 4. Materials and Methods

### 4.1. Materials and Reagents

JZGX was obtained from Guangdong Litai Pharmaceutical Co., Ltd. (Puning, China). Thirty-four chemical reference standards were obtained through the National Institute for Control of Biological and Pharmaceutical Products of China (Beijing, China), Chengnuo Biotech Co., Ltd. (Zhongshan, China), and Yuanye Biotech Co., Ltd. (Shanghai, China) (Table S6). MS grade methanol and acetonitrile were provided by Thermo Fisher Scientific Inc. (Fair Lawn, NJ, USA). MS grade formic acid was acquired from Sigma-Aldrich Co. (St Louis, MI, USA). The water was distilled and purified using a Milli-Q system (Millipore, Milford, MA, USA). All other reagents were of analytical grade.

### 4.2. UPLC-Q-TOF-MS/MS Analysis for JZGX Ingredient Identification

Five Chinese medicinal materials were extracted with deionized water for 1 h separately, and the extracts were concentrated to 50 mL. The crude drug dosage of the extracts was the same as that of JZGX Oral Liquids in Chinese Pharmacopoeia. Respectively, the JZGX samples (1 mL) and the extracts (1 mL) were accurately measured and diluted with deionized water to 50 mL. Then the diluents were ultrasonic-extracted (500 W, 40 kHz) for 15 min. At a concentration of 10 μg/mL for each compound, the mixed solutions of reference standards about saccharides were prepared with ddH_2_O for HILIC-MS analysis, and the other reference standards were dissolved in methanol for RPLC-MS identification. Before mass spectrometry detection, all test solutions were filtered through a 0.22 μm microporous filter.

UPLC-Q-TOF-MS/MS were composed with ultra-fast liquid chromatography (Shimadzu Corp., Kyoto, Japan) and quadrupole/time-of-flight mass spectrometry (Triple TOF 5600 plus, AB SCIEX, Foster City, CA, USA). HILIC separations were performed on a HILIC-B column (2.1 × 100 mm, 1.7 μm) and maintained at 40 °C. The mobile phase consisted of water (A) and acetonitrile (B). The following program carried out the elution: 70–53% B (0–10 min), 53–70% b (10–10.5 min), and 70% B (10.5–11 min), with the flow rate kept at 0.2 mL/min. The injection volume was 5 μL; RPLC separations were operated on a Kinetex C18 column (3.0 × 150 mm, 2.6 μm) and sustained at 40 °C with the flow rate kept at 0.3 mL/min. The mobile phase consisted of water containing 0.1% aqueous formic acid (*v*/*v*) (A) and acetonitrile (B), which was applied to the gradient elution method, which was set as follows: 5–35% B (0–20 min), 35–100% B (20–23 min), 100% B (23–25 min). The injection volume was 2 μL.

MS/MS identification used an electrospray ionization (ESI) source, with the ion spray voltage 5500 V in positive and −4500 V in negative ion modes. Both in the negative and positive modes, the HILIC mass scan range was from *m/z* 50–1500, and the RPLC mass range was from *m/z* 100–1500. Both ion source gas 1 and gas 2 were 55 psi, while the curtain gas was adjusted to 35 psi. The ion source temperature was maintained at 550 °C. The declustering potential was 80 V. The collision energy was 30 eV with a spread of 15 eV. Nitrogen was used as the nebulizer and auxiliary gas. Data was acquired using Analyst^®^ TF 1.6 software (AB Sciex, Foster City, CA, USA) in the information-dependent acquisition mode. Data analysis was performed using PeakView 2.2 (AB Sciex, Foster City, CA, USA).

### 4.3. Construction of the JZGX Active Component and CAD-related Target Network

According to the results of JZGX component identification, the SwissADME Database (http://www.swissadme.ch/, accessed on 9 December 2023) was applied to screen JZGX active components under the criteria of high GI absorption and drug likeness, including at least two yes. The prediction of potential targets related to the active ingredients of JZGX was obtained on the SwissTargetPrediction Database (http://www.swisstargetprediction.ch/, accessed on 9 December 2023). Cytoscape (Version 3.9.1) was applied to visualize the network between the active components and target genes.

Data mining for the treatment targets of CAD was conducted from open-source databases, putting in the keyword “coronary artery disease”. The databases included the Drugbank database (https://go.drugbank.com/, accessed on 9 December 2023), DisGeNET database (https://www.disgenet.org/, accessed on 9 December 2023), OMIM Database (https://www.omim.org/, accessed on 9 December 2023), and CTD database (https://ctdbase.org/, accessed on 9 December 2023). The targets corresponding to JZGX chemical components and related to CAD were unified as Gene Symbols using the Uniprot database (https://www.uniprot.org/, accessed on 9 December 2023). Selecting the overlaps as the JZGX potential therapeutic targets of CAD using a Venn diagram.

The STRING Database (Version 12.0, https://string-db.org/, accessed on 10 December 2023) was used to construct a PPI network about JZGX potential targets. The organism species were limited to “Homo sapiens” and the confidence score was > 0.9. The protein interaction results were imported into Cytoscape for the network topology analysis. Nodes exhibiting degree values twice the median of all network nodes, along with closeness centrality and betweenness centrality values above the median, were filtered as the principal target genes.

The GO and KEGG pathway analyses were conducted on the Metascape Database (Version 3.5.20240101) with a threshold of *p* < 0.05. Inputting the gene symbols of JZGX potential anti-CAD targets into the Metascape Database, setting the species to “Homo sapiens”, then selecting the functional annotation tool to analyze. The R language was used to visualize the enrichment outcomes.

### 4.4. Animals and Drug Administration

Animal protocols in this study were approved by the Ethics Committee of the School of Life Sciences, Sun Yat-sen University (Certificate No. SYSU-LS-IACUC-2023-0108).

Twenty-four male ApoE^−/−^ mice and eight wild-type C57BL/6J mice with the same genetic background (4-week-olds) were purchased from the Guangdong Medical Laboratory Animal Center (Certificate No. 44007200098265 and No. 44007200099011 respectively, Foshan, China). All animals were housed in a standardized pathogen-free area with ambient temperature (23~26 °C) and humidity (50~70%). In addition, they were allowed to acclimatize for 1 week under a 12-h light-dark cycle with free access to food and water.

ApoE^−/−^ mice (4-week-olds) were randomly divided into three distinct groups: (1) the model group (MOD, *n* = 8), (2) the atorvastatin treatment group (ATO, *n* = 8), and (3) the JZGX Oral Liquids treatment group (JZGX, *n* = 8). During the entire 24-week duration of the experiment, ApoE^−/−^ mice were provided with a high-fat diet (21% fat and 0.15% cholesterol), while C57BL/6J mice were fed a regular diet, serving as the control group (CON). At the age of eight weeks, the mice in the ATO group were subjected to oral administration of atorvastatin (10 mg/kg/d) using a gavage technique, while the mice in the JZGX group were orally supplied with JZGX Oral Liquids (20 g/kg/d) using the same method. The mice in the CON and MOD groups were given an equal volume of normal saline solution. All animals received constant daily intragastric injections throughout the 20 weeks. During the trial, the mice were fed ad libitum and were constantly monitored for changes in body weight, food consumption, water intake, and general physical condition. Upon completion of the experiment, the mice were subjected to an overnight fasting period. Subsequently, the mice were anesthetized using a 1% isoflurane aerosol, collected blood samples from the suborbital venous plexus, and then euthanized using carbon dioxide.

### 4.5. Histological Measurement

Heart tissues that included the aortic arch were surgically removed from the proximal aortic root to the branch of the iliac artery. These tissues were immersed in a 4% paraformaldehyde solution for 6 h. The aorta was separated carefully and fat tissue was removed from around it, and placed in a fixative (G1101, Wuhan servicebio technology CO., LTD., Wuhan, China) for more than 24 h. The aorta was immersed in 60% isopropyl alcohol (80109218, Sinopharm Chemical Reagent Co., Ltd., Shanghai, China) for 3 s and then immersed in Oil Red O staining solution (G1015, Wuhan servicebio technology CO., LTD., Wuhan, China) for 60 min at 37 °C in the dark. Immersing it in 60% isopropyl alcohol for differentiation. The differentiation time started from 1 min until the fat plaque in the lumen turned orange or bright red, and other parts were almost colorless. The heart was embedded in OCT and cut into serial 10-μm cross sections for HE, Oil Red O, and Masson staining to measure the lesion area and quantification of the lipid and collagen content, respectively.

Liver tissues were preserved in a 4% paraformaldehyde solution. Referring to the research [72], parts of liver tissues were sliced into 4-μm sections, dehydrated, and embedded in paraffin for HE staining; liver specimens embedded in OCT were cut into 10-μm sections using a frozen slicer for Oil Red O staining.

All experiments were performed according to the manufacturer’s instructions (Wuhan servicebio technology CO., LTD., Wuhan, China). The dimensions and extent of the lesion were assessed by a blind observer utilizing Image J software (Version 2.3.0). More detailed quantification methods using Image J software, refer to the article [73].

### 4.6. Detection of Biochemical Indexes

The blood was kept for 1 h at room temperature, followed by centrifugation for 10 min at 4000 rpm to attain serum. Part of the serum was used to detect the TC (A111-1-1), TG (A110-1-1), LDL-C (A113-1-1), HDL-C (A112-1-1), IL-6 (H007A), TNF-α (H052A), PGI2 (H214-1-2), TXA2 (H330-1-2), CK-MB (H197-1-2), cTnT (H149-4-2), AST (C010-2-1), and ALT (C009-2-1). All the kits were purchased from Nanjing Jiancheng Bio-engineering Institute (Nanjing, China) and the testing procedures were conducted according to the instructions. The remaining serum was stored at −80 °C.

### 4.7. Untargeted Serum Metabolomics Profiling

One hundred microliters of serum were thawed and mixed with 400 μL pre-cooled methanol/acetonitrile (1:1, *v*/*v*) containing 20 μg/mL of myristic acid-D27 obtained from Cambridge Isotope Laboratories (Andover, MA, USA) as an internal standard (IS). The mixture was then vortexed for 30 s and incubated at −20 °C for 1 h for complete protein precipitation. Centrifuge the samples at 4 °C for 20 min, 15,000× *g*. Take 200 μL supernatant and dry it with nitrogen at 37 °C. Add 80 μL methanal to reconstitute the residue. Centrifuge (4 °C, 15,000× *g*) for 10 min. The supernatant was taken and analyzed by UPLC-Q-TOF-MS/MS. Equal volumes (5 μL) from each sample were combined to create pooled quality control (QC) samples, which were injected into the monitor system during detection to assess and maintain stability.

The UPLC-Q-TOF-MS/MS system with ESI source employed in JZGX component identification above was also utilized here. Chromatographic separation was performed on ACQUITY UPLC BEH C18 (2.1 × 150 mm, 1.7 μm) and maintained at 40 °C with the flow rate kept at 0.3 mL/min. The mobile phase consisted of water containing 0.1% aqueous formic acid (*v*/*v*) (A) and acetonitrile (B). The elution methods are set as 5–78% B (0–6 min), 78–85% B (6–12 min), 85–100% B (12–15 min), and 100% B (15–18 min). The injection volume was 5 μL. The instrumental settings of Q-TOF-MS/MS and data acquisition methods were the same as the chemical composition collection previously described.

The mass spectrum data analysis was conducted by PeakView 2.2 and Markerview 1.2 software (AB Sciex, Foster City, CA, USA) for noise reduction, peak alignment, and identification. IS peak intensities were used to normalize. The orthogonal partial least squares discriminant (OPLS-DA) analysis was performed by SIMCA-P 14.1 (Umetrics AB, Umea, Sweden). The differential feature metabolites were screened by VIP > 1 and *p* < 0.05 (significance test). These metabolites were identified via mass fragmentation and compared with databases including HMDB (https://hmdb.ca/, accessed on 2 January 2024) and METLIN Gen2 (https://massconsortium.com/, accessed on 2 January 2024). MetaboAnalyst 6.0 (https://www.metaboanalyst.ca/MetaboAnalyst/, accessed on 3 January 2024) was used for the metabolic pathway cluster analysis.

### 4.8. Western Blotting

The liver tissues of mice were cut up to 20 mg and ground to homogenate with 200 μL RIPA lysis solution. The lysate was centrifuged at 5 min, 14,000× *g*, and the supernatant was isolated. The BCA protein concentration assay kit (P0009,Beyotime Biotechnology, Shanghai, China) was applied to determine the total protein concentration in the samples. The supernatants were mixed with protein loading buffer (P0015L, Beyotime Biotechnology, Shanghai, China) and incubated for 10 min at 100 °C. After SDS-PAGE gel electrophoresis, the proteins were transferred to PVDF membranes, incubated in the blocking buffer for 1 h, and washed with TBST (HE091, Genstar Biosolutions Co., Ltd., Beijing, China), followed by incubation with the primary antibody overnight at 4 °C. The primary antibodies, including PPARα (A24835, ABclonal Technology, Woburn, MA, USA), PPARγ (2443, Cell Signaling Technology, Inc., Danvers, MA, USA), SREBP1 (sc-365513), FASN (sc-48357), PTGS2 (sc-376861), CYP3A (sc-365415), and β-Actin (sc-47778), were purchased from Santa Cruz Biotechnology, Inc. (Dallas, Texas, USA). The diluted secondary antibodies (Mouse0000486440, Rabbit0000573275, Promega Corporation, Madison, WI, USA) were incubated at room temperature, and the membrane signals were visualized with an ECL solution (P00118FM, Beyotime Biotechnology, Shanghai, China). The relative expression of the target protein was analyzed with Image J software and calculated using β-Actin as the internal reference.

### 4.9. Molecular Docking

The active components and key targets were determined by the molecular docking analysis. Search the structures of each chemical on the Pubchem website (https://pubch-em.ncbi.nlm.nih.gov/, accessed on 1 March 2024) and download the “sdf” files of its 2D structure. The ChemBio3D software (Version 18.1) was used to convert 2D structures into 3D structures and performed energy minimization optimization processing. The 3D structures were then saved as “mol2” format files. Searching the best crystal structures of each protein target through the Protein Data Bank Database (PDB, https://www.pdbus.org/, accessed on 2 March 2024), which were saved as “pdb” format files. Converting the “mol2” format files of the chemical compositions into “pdbqt” format files through Autodock software (Version 1.5.7) processing. The PyMOL software (Version 2.6) was used to dehydrate and remove molecular ligands from the above protein crystal structures. Autodock software was employed to hydrogenate them and export them in “pdbqt” format. Finally, the Autodock Vina was applied to molecular docking of the above chemical components and protein targets, and the docking results were visualized through PyMOL software. The greater the absolute values of the docking binding energy, the better the binding effect between the ligands and the receptors.

### 4.10. Statistical Analysis

Statistical analysis was performed as described with minor modifications using SPSS statistical software (Version 22.0, SPSS. Inc, Chicago, IL, USA) and GraphPad Prism 9 (San Diego, CA, USA). The data were presented as mean values with a standard deviation (±SD). One-way analysis of variance (ANOVA), Student’s *t*-test, and Dunnett’s multiple comparisons were used to compare the results among groups. Differences were considered statistically significant at *p* < 0.05 or *p* < 0.01.

## 5. Conclusions

JZGX included 107 chemical components, and its treatment could impede atherosclerotic CAD progression via rewiring the lipid metabolism with the lessened formation of plaque in the coronary arteries, serum lipid levels, and deposition of hepatic lipids. PPAR-related proteins and the corresponding fatty acid β-oxidation metabolism were activated by JZGX, which was also proved by the enhanced carnitine transportation and the TCA cycle. JZGX was proved to be an ideal anti-CAD Chinese herbal formula and suggested as a potential solution for other lipid metabolism vasculopathy. In the next phase of the research, a quantitative analysis of JZGX chemical components and their in vivo metabolism will be conducted to provide a more comprehensive understanding of the mechanisms by which JZGX treats atherosclerotic CAD.

## Data Availability

Data is contained within the article and Supplementary Materials.

## Supplementary Materials

The following supporting information can be downloaded at: https://www.mdpi.com/article/10.3390/ph17060784/s1, Table S1: Identification of chemical compounds in JZGX Oral Liquids by UPLC-Q-TOF-MS/MS; Table S2: Potentially 39 active ingredients of JZGX; Table S3: The 37 key targets of JZGX in treating cardiovascular diseases; Table S4: The 107 differential metabolites significantly regulated by JZGX in serum metabolomic analysis; Table S5: Molecular docking of 39 active ingredients and PPARs; Table S6: The general information of reference standards; Figure S1: The permutation test on the OPLS-DA of the CON and MOD groups in the negative (A) and positive (B) modes, and the MOD and JZGX groups in the negative (C) and positive (D) mode.

## References

[1] Ralapanawa U., Sivakanesan R. (2021). Epidemiology and the magnitude of coronary artery disease and acute coronary syndrome: A narrative review. J. Epidemiol. Glob. Health.

[2] The Writing Committee of the Report on Cardiovascular Health and Diseases in China (2023). Report on Cardiovascular Health and Diseases in China 2022: An Updated Summary. Biomed. Environ. Sci..

[3] Davies M.J., Thomas A.C. (1985). Plaque fissuring—The cause of acute myocardial infarction, sudden ischaemic death, and crescendo angina. Br. Heart J..

[4] Björkegren J.L., Lusis A.J. (2022). Atherosclerosis: Recent developments. Cell.

[5] Frostegård J. (2013). Immunity, atherosclerosis and cardiovascular disease. BMC Med..

[6] Pflieger M., Winslow B.T., Mills K., Dauber I.M. (2011). Medical management of stable coronary artery disease. Am. Fam. Physician.

[7] Thompson P.D., Panza G., Zaleski A., Taylor B. (2016). Statin-associated side effects. J. Am. Coll. Cardiol..

[8] Tsui L., Ye P., Xu S., Lin Y., Chen B., Chen S., Cheng R. (2023). Adverse drug reactions of statin therapy in China from 1989 to 2019: A national database analysis. Eur. J. Hosp. Pharm..

[9] Pan S., Chen S., Dong H., Yu Z., Dong J., Long Z., Fong W., Han Y., Ko K. (2011). New perspectives on Chinese herbal medicine (Zhong-Yao) research and development. Evid. Based Complement. Alternat Med..

[10] Jiang Y., Zhao Q., Li L., Huang S., Yi S., Hu Z. (2022). Effect of traditional Chinese medicine on the cardiovascular diseases. Front. Pharmacol..

[11] Li Z., Xu S., Liu P. (2018). Salvia miltiorrhizaBurge (Danshen): A golden herbal medicine in cardiovascular therapeutics. Acta Pharmacol. Sin..

[12] Xie P., Cui L., Shan Y., Kang W. (2017). Antithrombotic effect and mechanism of radix paeoniae rubra. Biomed. Res. Int..

[13] Chen Z., Zhang C., Gao F., Fu Q., Fu C., He Y., Zhang J. (2018). A systematic review on the rhizome of Ligusticum chuanxiong Hort. (Chuanxiong). Food Chem. Toxicol..

[14] Tu Y., Xue Y., Guo D., Sun L., Guo M. (2015). Carthami flos: A review of its ethnopharmacology, pharmacology and clinical applications. Rev. Bras. Farmacogn..

[15] Zhao X., Wang C., Meng H., Yu Z., Yang M., Wei J. (2020). Dalbergia odorifera: A review of its traditional uses, phytochemistry, pharmacology, and quality control. J. Ethnopharmacol..

[16] Xin W., Zi-Yi W., Zheng J.-H., Shao L. (2021). TCM network pharmacology: A new trend towards combining computational, experimental and clinical approaches. Chin. J. Nat. Med..

[17] Zhao S., Liu Z., Wang M., He D., Liu L., Shu Y., Song Z., Li H., Liu Y., Lu A. (2018). Anti-inflammatory effects of Zhishi and Zhiqiao revealed by network pharmacology integrated with molecular mechanism and metabolomics studies. Phytomedicine.

[18] Hedayatnia M., Asadi Z., Zare-Feyzabadi R., Yaghooti-Khorasani M., Ghazizadeh H., Ghaffarian-Zirak R., Nosrati-Tirkani A., Mohammadi-Bajgiran M., Rohban M., Sadabadi F. (2020). Dyslipidemia and cardiovascular disease risk among the MASHAD study population. Lipids Health Dis..

[19] Hartman J., Frishman W.H. (2014). Inflammation and atherosclerosis: A review of the role of interleukin-6 in the development of atherosclerosis and the potential for targeted drug therapy. Cardiol. Rev..

[20] Herrmann S.-M., Ricard S., Nicaud V., Mallet C., Arveiler D., Evans A., Ruidavets J.B., Luc G., Bara L., Parra H.J. (1998). Polymorphisms of the tumour necrosis factor-α gene, coronary heart disease and obesity. Eur. J. Clin. Invest..

[21] Gryglewski R., Dembínska-Kieć A., Korbut R. (1978). A possible role of thromboxane A2 (TXA2) and prostacyclin (PGI2) in circulation. Acta Biol. Med. Ger..

[22] Peela J., Jarari A., Hai A., Rawal A., Kolla S., Sreekumar S., Khurana L., Sidhanathi N. (2010). Cardiac biomarkers: The troponins and CK-MB. J. Medical Biomed. Sci..

[23] Nguyen P., Leray V., Diez M., Serisier S., Le Bloc’h J., Siliart B., Dumon H. (2008). Liver lipid metabolism. J. Anim. Physiol. Anim. Nutr..

[24] Galiero R., Caturano A., Vetrano E., Cesaro A., Rinaldi L., Salvatore T., Marfella R., Sardu C., Moscarella E., Gragnano F. (2021). Pathophysiological mechanisms and clinical evidence of relationship between Nonalcoholic fatty liver disease (NAFLD) and cardiovascular disease. Rev. Cardiovasc. Med..

[25] Kew M.C. (2000). Serum aminotransferase concentration as evidence of hepatocellular damage. Lancet.

[26] Kunutsor S.K., Apekey T.A., Khan H. (2014). Liver enzymes and risk of cardiovascular disease in the general population: A meta-analysis of prospective cohort studies. Atherosclerosis.

[27] Pinto R.C. (2017). Chemometrics methods and strategies in metabolomics. Metabolomics: From Fundamentals to Clinical Applications.

[28] Zandbergen F., Plutzky J. (2007). PPARα in atherosclerosis and inflammation. Biochim. Biophys. Acta.

[29] Han L., Shen W.-J., Bittner S., Kraemer F.B., Azhar S. (2017). PPARs: Regulators of metabolism and as therapeutic targets in cardiovascular disease. Part II: PPAR-β/δ and PPAR-γ. Future Cardiol..

[30] Nordestgaard B.G., Wootton R., Lewis B. (1995). Selective retention of VLDL, IDL, and LDL in the arterial intima of genetically hyperlipidemic rabbits in vivo. Molecular size as a determinant of fractional loss from the intima-inner media. Arterioscler. Thromb. Vasc. Biol..

[31] McLaren J.E., Michael D.R., Ashlin T.G., Ramji D.P. (2011). Cytokines, macrophage lipid metabolism and foam cells: Implications for cardiovascular disease therapy. Prog. Lipid Res..

[32] Ponziani F.R., Pecere S., Gasbarrini A., Ojetti V. (2015). Physiology and pathophysiology of liver lipid metabolism. Expert. Rev. Gastroenterol. Hepatol..

[33] Huang N.K., Bůžková P., Matthan N.R., Djoussé L., Hirsch C.H., Kizer J.R., Longstreth W., Mukamal K.J., Lichtenstein A.H. (2021). Associations of serum nonesterified fatty acids with coronary heart disease mortality and nonfatal myocardial infarction: The CHS (cardiovascular health study) cohort. J. Am. Heart Assoc..

[34] Nomura S.O., Karger A.B., Weir N.L., Duprez D.A., Tsai M.Y. (2020). Free fatty acids, cardiovascular disease, and mortality in the Multi-Ethnic Study of Atherosclerosis. J. Clin. Lipidol..

[35] Hennig B., Toborek M., McClain C.J. (2001). High-Energy Diets, Fatty Acids and Endothelial Cell Function: Implications for Atherosclerosis. J. Am. Coll. Nutr..

[36] Jira W., Spiteller G., Carson W., Schramm A. (1998). Strong increase in hydroxy fatty acids derived from linoleic acid in human low density lipoproteins of atherosclerotic patients. Chem. Phys. Lipids.

[37] Parthasarathy S., Litvinov D., Selvarajan K., Garelnabi M. (2008). Lipid peroxidation and decomposition—Conflicting roles in plaque vulnerability and stability. Biochim. Biophys. Acta.

[38] Levitan I., Volkov S., Subbaiah P.V. (2010). Oxidized LDL: Diversity, Patterns of Recognition, and Pathophysiology. Antioxid. Redox Signal.

[39] Kzhyshkowska J., Neyen C., Gordon S. (2012). Role of macrophage scavenger receptors in atherosclerosis. Immunobiology.

[40] Wang B., Wu L., Chen J., Dong L., Chen C., Wen Z., Hu J., Fleming I., Wang D.W. (2021). Metabolism pathways of arachidonic acids: Mechanisms and potential therapeutic targets. Signal Transduct. Target. Ther..

[41] Yu K., Bayona W., Kallen C.B., Harding H.P., Ravera C.P., McMahon G., Brown M., Lazar M.A. (1995). Differential Activation of Peroxisome Proliferator-activated Receptors by Eicosanoids. J. Biol. Chem..

[42] Poulsen L.l.C., Siersbæk M., Mandrup S. (2012). PPARs: Fatty acid sensors controlling metabolism. Semin. Cell Dev. Biol..

[43] Campbell W.B., Spitzbarth N., Gauthier K.M., Pfister S.L. (2003). 11,12,15-Trihydroxyeicosatrienoic acid mediates ACh-induced relaxations in rabbit aorta. Am. J. Physiol. Heart Circ. Physiol..

[44] Yun M.R., Lee J.Y., Park H.S., Heo H.J., Park J.Y., Bae S.S., Hong K.W., Sung S.M., Kim C.D. (2006). Oleic acid enhances vascular smooth muscle cell proliferation via phosphatidylinositol 3-kinase/Akt signaling pathway. Pharmacol. Res..

[45] Ma S., Yang D., Li D., Tang B., Yang Y. (2011). Oleic acid induces smooth muscle foam cell formation and enhances atherosclerotic lesion development via CD36. Lipids Health Dis..

[46] Lopaschuk G.D., Ussher J.R., Folmes C.D., Jaswal J.S., Stanley W.C. (2010). Myocardial fatty acid metabolism in health and disease. Physiol. Rev..

[47] Houten S.M., Wanders R.J.A. (2010). A general introduction to the biochemistry of mitochondrial fatty acid β-oxidation. J. Inherit. Metab. Dis..

[48] Kukharenko A., Brito A., Kozhevnikova M.V., Moskaleva N., Markin P.A., Bochkareva N., Korobkova E.O., Belenkov Y.N., Privalova E.V., Larcova E.V. (2020). Relationship between the plasma acylcarnitine profile and cardiometabolic risk factors in adults diagnosed with cardiovascular diseases. Clin. Chim. Acta.

[49] Alves-Bezerra M., Cohen D.E. (2017). Triglyceride Metabolism in the Liver. Compr. Physiol..

[50] Talayero B.G., Sacks F.M. (2011). The Role of Triglycerides in Atherosclerosis. Curr. Cardiol. Rep..

[51] Chatterjee S. (1998). Sphingolipids in atherosclerosis and vascular biology. Arterioscler. Thromb. Vasc. Biol..

[52] Merten M., Dong J.F., Lopez J.A., Thiagarajan P. (2001). Cholesterol sulfate: A new adhesive molecule for platelets. Circulation.

[53] Murphy C., Parini P., Wang J., Björkhem I., Eggertsen G., Gåfvels M. (2005). Cholic acid as key regulator of cholesterol synthesis, intestinal absorption and hepatic storage in mice. Biochim. Biophys. Acta.

[54] Chirala S.S., Wakil S.J. (2004). Structure and function of animal fatty acid synthase. Lipids.

[55] Semple R.K., Chatterjee V.K.K., O’Rahilly S. (2006). PPARγ and human metabolic disease. J. Clin. Investig..

[56] Janani C., Kumari B.R. (2015). *PPAR* gamma gene–a review. Diabetes Metab. Syndr..

[57] Siva D., Abinaya S., Rajesh D., Archunan G., Padmanabhan P., Gulyás B., Achiraman S. (2022). Mollification of doxorubicin (DOX)-mediated cardiotoxicity using conjugated chitosan nanoparticles with supplementation of propionic acid. Nanomaterials.

[58] Jiang C., Ting A.T., Seed B. (1998). PPAR-γ agonists inhibit production of monocyte inflammatory cytokines. Nature.

[59] Nagy L., Tontonoz P., Alvarez J.G., Chen H., Evans R.M. (1998). Oxidized LDL regulates macrophage gene expression through ligand activation of PPARγ. Cell.

[60] Chawla A., Boisvert W.A., Lee C.-H., Laffitte B.A., Barak Y., Joseph S.B., Liao D., Nagy L., Edwards P.A., Curtiss L.K. (2001). A PPARγ-LXR-ABCA1 pathway in macrophages is involved in cholesterol efflux and atherogenesis. Mol. Cell.

[61] Horton J.D., Goldstein J.L., Brown M.S. (2002). SREBPs: Activators of the complete program of cholesterol and fatty acid synthesis in the liver. J. Clin. Investig..

[62] Sekiya M., Yahagi N., Matsuzaka T., Takeuchi Y., Nakagawa Y., Takahashi H., Okazaki H., Iizuka Y., Ohashi K., Gotoda T. (2007). SREBP-1-independent regulation of lipogenic gene expression in adipocytes. J. Lipid Res..

[63] Latasa M.-J., Griffin M.J., Moon Y.S., Kang C., Sul H.S. (2003). Occupancy and function of the− 150 sterol regulatory element and− 65 E-box in nutritional regulation of the fatty acid synthase gene in living animals. Mol. Cell Biol..

[64] Gosmain Y., Dif N., Berbe V., Loizon E., Rieusset J., Vidal H., Lefai E. (2005). Regulation of SREBP-1 expression and transcriptional action on HKII and FAS genes during fasting and refeeding in rat tissues. J. Lipid Res..

[65] Schroeder F., Petrescu A.D., Huang H., Atshaves B.P., McIntosh A.L., Martin G.G., Hostetler H.A., Vespa A., Landrock D., Landrock K.K. (2008). Role of fatty acid binding proteins and long chain fatty acids in modulating nuclear receptors and gene transcription. Lipids.

[66] Klyushova L.S., Perepechaeva M.L., Grishanova A.Y. (2022). The Role of CYP3A in Health and Disease. Biomedicines.

[67] Hashimoto M., Kobayashi K., Watanabe M., Kazuki Y., Takehara S., Inaba A., Nitta S.I., Senda N., Oshimura M., Chiba K. (2013). Knockout of mouse *Cyp3a* gene enhances synthesis of cholesterol and bile acid in the liver. J. Lipid Res..

[68] Jamwal R., de la Monte S.M., Ogasawara K., Adusumalli S., Barlock B.B., Akhlaghi F. (2018). Nonalcoholic Fatty Liver Disease and Diabetes Are Associated with Decreased CYP3A4 Protein Expression and Activity in Human Liver. Mol. Pharm..

[69] Leclercq I.A., Farrell G.C., Schriemer R., Robertson G.R. (2002). Leptin is essential for the hepatic fibrogenic response to chronic liver injury. J. Hepatol..

[70] Funk C.D. (2001). Prostaglandins and leukotrienes: Advances in eicosanoid biology. Science.

[71] Chitturi S., Farrell G.C. (2001). Etiopathogenesis of nonalcoholic steatohepatitis. Semin. Liver Dis..

[72] Lu W., Mei J., Yang J., Wu Z., Liu J., Miao P., Chen Y., Wen Z., Zhao Z., Kong H. (2020). *ApoE* deficiency promotes non-alcoholic fatty liver disease in mice via impeding AMPK/mTOR mediated autophagy. Life Sci..

[73] Collins T.J. (2007). ImageJ for microscopy. Biotechniques.

