# Supplementary material for: Jingzhi Guanxin Oral Liquids Attenuate Atherosclerotic Coronary Heart Disease via Modulating Lipid Metabolism and PPAR-Related Targets
Source: Pharmaceuticals (Basel). 2024 Jun 14;17(6):784. doi: 10.3390/ph17060784 (PMC11206304; doi:10.3390/ph17060784)
Supplement: Supplementary file 1 [file pharmaceuticals-17-00784-s001.zip › pharmaceuticals-3007228-supplementary/Supplementary materials/Supplementary Materials.pdf]

Table S1. Identification of chemical compounds in JZGX Oral Liquids by UPLC-Q-TOF-MS/MS.

No.	Identification	Molecular Formula	Retention Time (min)	$[M+H]^+$ (Error，ppm)	$[M–H]^–$ (Error，ppm)	Fragment Ions in the Positive Ion Mode(*m/z*)	Fragment Ions in the Negative Ion Mode(*m/z*)	Source
1	D-Glucose [a,b,c]	$C_6H_{12}O_6$	2.54	ND	179.0567<br>(3.1)	ND	89.0247[M–H–$C_3H_6O_3$]$^–$,<br>71.0139[M–H–$C_3H_6O_3$–H_2O]$^–$,<br>59.0138[M–H–$C_4H_8O_4$]$^–$	DS、CS、CX、HH
2	D-Mannitol [a,b,c]	$C_6H_{14}O_6$	2.67	ND	181.0726<br>(0.2)	ND	163.0619[M–H–H_2O]$^–$,<br>89.0242[M–H–$C_3H_8O_3$]$^–$,<br>71.0141[M–H–$C_3H_8O_3$–H_2O]$^–$,<br>59.0138[M–H–$C_4H_{10}O_4$]$^–$	DS、CS、CX、HH
3	Sucrose [a,b,c]	$C_{12}H_{22}O_{11}$	3.00	343.1232<br>(–0.8)	341.1092<br>(0.9)	180.0836[M+H–$C_6H_{11}O_5$]$^+$,<br>162.0754[M+H–$C_6H_{13}O_6$]$^+$,<br>144.0654[M+H–$C_6H_{13}O_6$–H_2O]$^+$	179.0568[M–H–$C_6H_{10}O_5$]$^–$,<br>161.0467[M–H–$C_6H_{12}O_6$]$^–$,<br>89.0243[M–H–$C_9H_{16}O_8$]$^–$,<br>71.0140[M–H–$C_9H_{16}O_8$–H_2O]$^–$	DS、CS、CX、HH
4	Raffinose [a,b,c]	$C_{18}H_{32}O_{16}$	3.83	ND	503.1622<br>(0.9)	ND	341.1096[M–H–$C_6H_{10}O_5$]$^–$,<br>221.0678[M–H–$C_{10}H_{18}O_9$]$^–$,<br>179.0574[M–H–$C_{12}H_{20}O_{10}$]$^–$,<br>89.0247[M–H–$C_{15}H_{26}O_{13}$]$^–$	DS、CS、CX
5	Trisaccharide [c]	$C_{18}H_{32}O_{16}$	4.60	ND	503.1620<br>(0.5)	ND	383.1200[M–H–$C_4H_8O_4$]$^–$,<br>341.1093[M–H–$C_6H_{10}O_5$]$^–$,<br>179.0566[M–H–$C_{12}H_{20}O_{10}$]$^–$	DS、CS
6	D-Stachyose [a,b,c]	$C_{24}H_{42}O_{21}$	5.02	ND	665.2152<br>(1.0)	ND	485.1526[M–H–$C_6H_{12}O_6$]$^–$,<br>341.1099[M–H–$C_{12}H_{20}O_{10}$]$^–$,<br>179.0570[M–H–$C_{18}H_{30}O_{15}$]$^–$	DS、CS
7	Pentose [c]	$C_{30}H_{52}O_{26}$	6.26	ND	827.2683<br>(1.1)	ND	827.2702[M–H]$^–$	DS
8	2'-Deoxyadenosine [c]	$C_{10}H_{13}N_5O_3$	8.46	252.1074<br>(–6.7)	250.0943<br>(–0.9)	ND	160.0620[M–H–$C_4H_{10}O_2$]$^–$,<br>88.0408[M–H–$C_6H_4N_5O$]$^–$	HH
9	L-Arginine [a,b,d]	$C_6H_{14}N_4O_2$	1.07	175.1187<br>(–1.5)	173.1045<br>(0.6)	130.0949[M+H–CH_4N_2]$^+$,<br>116.0681[M+H–CH_5N_3]$^+$	131.0823[M–H–CH_2N_2]$^–$	CS、CX、HH
10	D-Gluconic acid [b,d]	$C_6H_{12}O_7$	1.22	ND	195.0515<br>(2.5)	ND	159.0304[M–H–2H_2O]$^–$<br>129.0190[M–H–3H_2O]$^–$	DS、CS、CX、HH
11	D-Glucaric acid [b,d]	$C_6H_{10}O_8$	1.24	ND	209.0308<br>(2.3)	ND	191.0200[M–H–H_2O]$^–$,<br>133.0136[M–H–$C_2H_4O_3$]$^–$,<br>85.0287[M–H–$C_2H_4O_6$]$^–$,	DS、CS、HH
12	Malic acid [a,b,d]	$C_4H_6O_5$	1.42	ND	133.0143<br>(0.7)	ND	115.0038[M–H–H_2O]$^–$,<br>89.0241[M–H–CO_2]$^–$	DS、CS、CX、HH
13	Citric acid [a,b,d]	$C_6H_8O_7$	1.93	ND	191.0203<br>(3.1)	ND	111.0083[M–H–2H_2O-COOH]$^–$,<br>87.0081[M–H–$C_2H_4O_2$-COOH]$^–$	DS、CS、CX、HH
14	2'-O-Methyluridine [d]	$C_{10}H_{14}N_2O_6$	1.96	259.0924<br>(–0.2)	257.0782<br>(1.1)	130.0490[M+H–$C_4H_3N_2O_2$–H_2O]$^+$,<br>84.0436[M+H–$C_4H_3N_2O_2$–2CH_4O]$^+$	128.0352[M–H–$C_4H_3N_2O_2$–H_2O]$^–$	DS
15	Uridine [b,d]	$C_9H_{12}N_2O_6$	1.99	245.0764<br>(–1.8)	243.0626<br>(1.5)	ND	200.0555[M–H–CHNO]$^–$,<br>110.0250[M–H–$C_5H_9O_4$]$^–$,<br>82.0323[M–H–$C_6H_{12}NO_4$]$^–$,	DS、CX、HH

16	Adenosine [a,b,d]	$C_{10}H_{13}N_5O_4$	2.09	268.1040 (0)	ND	136.0613[M+H–$C_5H_8O_4$]$^+$, 119.0353[M+H–$C_5H_8O_4$–NH_3]$^+$	ND	CS、CX、HH
17	Gallic acid [a,b,d]	$C_7H_6O_5$	2.34	171.0287 (–0.8)	169.0145 (1.3)	153.0182[M+H–H_2O]$^+$, 109.0273[M+H–H_2O–CO_2]$^+$, 81.0328[M+H–$C_3H_6O_3$]$^+$	125.0242[M–H–CO_2]$^-$, 97.0292[M–H–$C_3H_4O_2$]$^-$, 81.0342 [M–H–$C_3H_4O_3$]$^-$	CS
18	1-O-β-D-Glucopyranosylpaeonisuffrone [d]	$C_{16}H_{24}O_9$	2.45	361.1499 (1.2)	ND	199.1067[M+H–$C_6H_{10}O_5$]$^+$, 181.0852[M+H–$C_6H_{12}O_6$]$^+$, 163.0747[M+H–$C_{10}H_{14}O_4$]$^+$, 135.0796[M+H–$C_{11}H_{14}O_5$]$^+$	ND	CS
19	L-Phenylalanine [a,b,d]	$C_9H_{11}NO_2$	2.76	166.0860 (–1.8)	164.0719 (1.0)	120.0796[M+H–CH_2O_2]$^+$, 103.0530[M+H–CH_2O_2–NH_3]$^+$, 91.0537[M+H–$C_2H_5NO_2$]$^+$	147.0441[M–H–NH_3]$^-$, 103.0544[M–H–NH_3–CO_2]$^-$	DS、CS、CX、HH
20	Danshensu [a,b,d]	$C_9H_{10}O_5$	3.21	199.0599 (–0.9)	197.0463 (3.5)	ND	179.0362[M–H–H_2O]$^-$, 135.0457[M–H–H_2O–CO_2]$^-$, 123.0454[M–H–$C_2H_2O_3$]$^-$, 109.0296[M–H–$C_3H_4O_3$]$^-$	DS
21	Protocatechuic acid [a,b,d]	$C_7H_6O_4$	3.56	155.0330 (–3.9)	153.0196 (1.5)	137.0220[M+H–H_2O]$^+$, 111.0429[M+H–CH_2O_2]$^+$, 93.0328[M+H–H_2O–CO_2]$^+$	109.0296[M–H–CO_2]$^-$, 91.0187[M–H–CO_2–H_2O]$^-$	DS、CX
22	Methyl gallate [d]	$C_8H_8O_5$	4.24	ND	183.0304 (1.9)	ND	168.0058[M–H–CH_3]$^-$, 124.0162[M–H–$C_2H_3O_2$]$^-$, 95.0113[M–H–$C_3H_4O_3$]$^-$	CS
23	6-Hydroxyquercetin-3-O-β-D-sophoroside-7-O-β-D-glucuronide [d]	$C_{33}H_{38}O_{24}$	4.46	819.1827 (0.2)	817.1698 (1.6)	657.1294[M+H–$C_6H_{10}O_5$]$^+$, 495.0768[M+H–$C_{12}H_{20}O_{10}$]$^+$, 319.0446[M+H–$C_{12}H_{20}O_{10}$–$C_6H_8O_6$]$^+$	ND	HH
24	Protocatechualdehyde [a,b,d]	$C_7H_6O_3$	4.92	139.0382 (–4.7)	137.0248 (3.1)	111.0431[M+H–CO]$^+$, 93.0326[M+H–CO–H_2O]$^+$	108.0218[M–H–CHO]$^-$, 92.0267[M–H–CHO–O]$^-$	DS、CS、CX
25	Oxypaeoniflorin [a,d]	$C_{23}H_{28}O_{12}$	5.21	497.1655 (0.4)	495.1512 (0.7)	335.1129[M+H–$C_6H_{10}O_5$]$^+$ 197.0804[M+H–$C_6H_{10}O_5$–$C_7H_6O_3$]$^+$ 121.0275[M+H–$C_{16}H_{24}O_{10}$]$^+$	137.0243[M–H–$C_{16}H_{22}O_9$]$^-$	CS
26	6-Hydroxykaempferol-3,6-O-β-D-glucoside-7-O-β-D-glucuronide [d]	$C_{33}H_{38}O_{23}$	5.39	803.1877 (0.1)	801.1750 (2.3)	641.1326[M+H–$C_6H_{10}O_5$]$^+$, 479.0800[M+H–2$C_6H_{10}O_5$]$^+$, 303.0493[M+H–2$C_6H_{10}O_5$–$C_6H_8O_6$]$^+$	625.1431[M–H–$C_6H_8O_6$]$^-$, 463.0894[M–H–$C_6H_8O_6$–$C_6H_{10}O_5$]$^-$	HH
27	6-Hydrokaempferol-3,6,7-triglucoside [d]	$C_{33}H_{40}O_{22}$	5.48	789.2086 (0.2)	787.1958 (2.2)	627.1534[M+H–$C_6H_{10}O_5$]$^+$ 465.1031[M+H–2$C_6H_{10}O_5$]$^+$ 303.0490[M+H–$C_{15}H_{16}O_8$–$C_6H_{10}O_5$]$^+$	625.1439[M–H–$C_6H_{10}O_5$]$^-$, 463.0909[M–H–2$C_6H_{10}O_5$]$^-$	HH
28	Chlorogenic acid [a,b,d]	$C_{16}H_{18}O_9$	5.68	355.1026 (0.8)	353.0881 (0.9)	163.0381[M+H–$C_7H_{12}O_6$]$^+$, 145.0278[M+H–$C_7H_{12}O_6$–H_2O]$^+$	191.0567[M–H–$C_9H_6O_3$]$^-$	CX、HH
29	1,2,3,4-Tetrahydro-3-carboxy-2-carboline [d]	$C_{12}H_{12}N_2O_2$	5.75	217.0969 (–1.0)	215.0833 (3.1)	144.0793[M+H–$C_3H_5O_2$]$^+$	171.0918[M–H–CO_2]$^-$, 142.0666[M–H–$C_2H_3NO_2$]$^-$, 116.0506[M–H–$C_4H_5NO_2$]$^-$, 92.0495[M–H–$C_6H_5NO_2$]$^-$	HH
30	Hydroxysafflor yellow A [a,b,d]	$C_{27}H_{32}O_{16}$	5.97	613.1766 (0.5)	611.1628 (1.7)	451.1215[M+H–$C_6H_{10}O_5$]$^+$, 433.1116[M+H–$C_6H_{10}O_5$–H_2O]$^+$, 355.0808[M+H–2$C_4H_8O_4$–H_2O]$^+$, 313.0657[M+H–2$C_6H_{10}O_5$]$^+$	491.1218[M–H–$C_4H_8O_4$]$^-$, 473.1116[M–H–$C_4H_8O_4$–H_2O]$^-$, 403.1053[M–H–$C_4H_8O_4$–H_2O–C_2H_2O–CO]$^-$, 325.0742[M–H–2$C_4H_8O_4$–H_2O–CO]$^-$	HH

31	Phthalic acid [b,d]	$C_8H_6O_4$	6.16	167.0337 (–1.4)	165.0199 (3.2)	$149.0225[M+H–H_2O]^+$, $121.0296[M+H–CH_2O_2]^+$, $93.0426[M+H–C_3H_6O_2]^+$	$121.0293[M–H–CO_2]^–$	CX
32	Caffeic acid [a,b,d]	$C_9H_8O_4$	6.35	181.0494 (–0.8)	179.0355 (3.0)	$163.0387[M+H–H_2O]^+$, $145.0275[M+H–2H_2O]]^+$, $135.0428[M+H–CH_2O_2]^+$, $117.0323[M+H–CH_2O_2–H_2O]^+$	$135.0450[M–H–CO_2]^–$	DS、CX、HH
33	6-Hydroxykaempferol-3-O-β-D-glucoside-7-O-β-D-glucuronide [d]	$C_{27}H_{28}O_{18}$	6.44	641.1344 (–0.7)	639.1216 (1.3)	$479.0812[M+H–C_6H_{10}O_5]^+$, $303.0498[M+H–C_6H_{10}O_5–C_6H_8O_6]^+$	$463.0882[M–H–C_6H_8O_6]^–$, $301.0355[M–H–C_6H_8O_6–C_6H_{10}O_5]^–$	HH
34	6-Hydroxyapigenin-6-O-β-D-glucoside-7-O-β-D-glucuronide [d]	$C_{27}H_{28}O_{17}$	6.96	625.1392 (–1.2)	623.1269 (2.4)	$463.0852[M+H–C_6H_{10}O_5]^+$, $287.0539[M+H–C_6H_{10}O_5–C_6H_8O_6]^+$	$447.0936[M–H–C_6H_8O_6]^–$, $284.0305[M–H–C_6H_8O_6–C_6H_{11}O_5]^–$, $113.0217[M–H–C_{21}H_{19}O_{11}–CHO–H_2O]^–$	HH
35	Albiflorin [a,b,d]	$C_{23}H_{28}O_{11}$	7.96	481.1707 (0.6)	479.1560 (0.2)	$319.1163[M+H–C_6H_{10}O_5]^+$, $301.1061[M+H–C_6H_{12}O_6]^+$, $197.0794[M+H–C_6H_{12}O_6–C_7H_4O]^+$, $105.0315[M+H–C_{16}H_{24}O_{10}]^+$	ND	CS
36	Vitexin [b,d]	$C_{21}H_{20}O_{10}$	8.18	433.1131 (0.4)	ND	$397.0910[M+H–2H_2O]^+$, $337.0722[M+H–C_6H_8O]^+$, $313.0705[M+H–C_4H_8O_4]^+$, $283.0580[M–H–C_5H_{10}O_5]^–$	ND	HH
37	6-Hydrokaempferol-3-O-β-rutinoside-6-O-β-D-glucoside [d]	$C_{33}H_{40}O_{21}$	8.28	773.2144 (1.0)	771.2009 (1.6)	$627.1550[M+H–C_6H_{10}O_4]^+$, $465.1022[M+H–C_{12}H_{20}O_9]^+$, $303.0501[M+H–C_{12}H_{20}O_9–C_6H_{12}O_5]^+$	$609.1484[M–H–C_6H_{10}O_5]^–$	HH
38	Vanillin [a,d]	$C_8H_8O_3$	8.32	153.0539 (–2.8)	151.0402 (1.1)	$125.0585[M+H–CO]^+$, $93.0324[M+H–CHO–CH_3O]^+$	ND	CX
39	Quercetin 3,7-diglucoside [d]	$C_{27}H_{30}O_{17}$	8.40	627.1563 (1.2)	625.1420 (1.5)	$465.1020[M+H–C_6H_{10}O_5]^+$, $303.0496[M+H–2C_6H_{10}O_5]^+$	$463.0889[M–H–C_6H_{10}O_5]^–$, $301.0358[M–H–2C_6H_{10}O_5]^–$	HH
40	*p*-Coumaric acid [a,b,d]	$C_9H_8O_3$	8.51	165.0540 (–2.4)	163.0408 (4.3)	$147.0410[M+H–H_2O]^+$, $123.0412[M+H–C_2H_2O]^+$, $119.0467[M+H–CH_2O_2]^+$, $95.0477[M+H–C_4H_6O]^+$, $91.0531[M+H–C_3H_6O_2]^+$	$119.0501[M–H–CO_2]^–$, $93.0342[M–H–C_3H_2O_2]^–$	HH
41	Kaempferol-3-β-D-glucoside-7-β-D-glucuronide [d]	$C_{27}H_{28}O_{17}$	8.64	625.1398 (–0.1)	623.1260 (1.0)	$463.0857[M+H–C_6H_{10}O_5]^+$, $287.0543[M+H–C_6H_{10}O_5–C_6H_8O_6]^+$	$447.0934[M–H–C_6H_8O_6]^–$, $285.0411[M–H–C_{15}H_{14}O_9]^–$	HH
42	Paeoniflorin [a,b,d]	$C_{23}H_{28}O_{11}$	8.65	481.1738 (0.7)	479.1561 (0.2)	$179.0701[M+H–C_{17}H_{18}O_5]^+$, $151.0751[M+H–C_6H_{11}O_6–C_8H_7O_2–H_2O]^+$, $135.0803[M+H–C_{15}H_{22}O_9]^+$	ND	CS
43	Kaempferol-3-O-β-sophoroside [d]	$C_{27}H_{30}O_{16}$	8.68	611.1607 (0.1)	609.1462 (0.2)	ND	$446.0840[M–H–C_6H_{11}O_5]^–$, $285.0399[M–H–C_{12}H_{20}O_{10}]^–$, $129.0193[M–H–C_{21}H_{16}O_{11}–2H_2O]^–$	HH
44	*p*-Coumaric acid glucoside [b,d]	$C_{15}H_{18}O_8$	8.79	327.1076 (0.4)	325.0933 (0.7)	$228.9771[M+H–C_5H_7O_2]^+$, $165.0541[M+H–C_9H_6O_3]^+$, $147.0432[M+H–C_6H_{10}O_5–H_2O]^+$, $119.0481[M+H–C_6H_{10}O_5–CH_2O_2]^+$	$163.0405[M–H–C_9H_6O_3]^–$, $119.0501[M–H–CO_2–C_6H_{10}O_5]^–$	HH

45	Quercetin-3-O-β-D-glucopyranoside [d]	$C_{21}H_{20}O_{12}$	9.52	465.1035<br>(1.0)	463.0886<br>(1.0)	$303.0506[M+H-C_6H_{10}O_5]^+$	$301.0360[M-H-C_6H_{10}O_5]^-$	HH
46	Ferulic acid [a,b,d]	$C_{10}H_{10}O_4$	9.82	195.0649<br>(–1.2)	193.0512<br>(2.9)	$177.0996[M+H-H_2O]^+$,<br>$149.1044[M+H-CH_2O_2]^+$,<br>$145.0259[M+H-CH_3O-H_2O]^+$,<br>$121.0654[M+H-C_3H_6O_2]^+$	$178.0270[M-H-CH_3]^-$,<br>$149.0588[M-H-CO_2]^-$,<br>$134.0375[M-H-CH_3-CO_2]^-$	CX
47	Isocarthamin [d]	$C_{21}H_{22}O_{11}$	10.07	451.1232<br>(–0.6)	449.1091<br>(0.3)	$289.0710[M+H-C_6H_{10}O_5]^+$,<br>$169.0121[M+H-C_6H_{10}O_5-C_8H_8O]^+$	$403.1614[M-H-H_2O-CO]^-$,<br>$287.0556[M-H-C_6H_{10}O_5]^-$,<br>$161.0446[M-H-C_{15}H_{12}O_6]^-$	HH
48	Rutin [a,b,d]	$C_{27}H_{30}O_{16}$	10.13	611.1610<br>(0.5)	609.1468<br>(1.2)	$465.1027[M+H-C_6H_{10}O_4]^+$,<br>$303.0500[M+H-C_{12}H_{20}O_9]^+$	$300.0278[M-H-C_{12}H_{21}O_9]^-$	HH
49	Salvianolic acid U [d]	$C_{27}H_{22}O_{12}$	10.32	539.1187<br>(0.6)	537.1041<br>(0.4)	$323.0546[M+H-C_9H_{10}O_5-H_2O]^+$,<br>$297.0753[M+H-C_9H_{10}O_5-CH_2O_2]^+$,<br>$279.0645[M+H-C_9H_{10}O_5-CO_2-H_2O]^+$,<br>$181.0486[M+H-C_{18}H_{14}O_8]^+$	$493.1155[M-H-CO_2]^-$,<br>$313.0735[M-H-CO_2-C_9H_8O_4]^-$,<br>$295.0624[M-H-CO_2-C_9H_{10}O_5]^-$	DS
50	Tinctormine [d]	$C_{27}H_{31}NO_{14}$	10.39	594.1817<br>(0)	592.1673<br>(0.3)	$432.1292[M+H-C_6H_{10}O_5]^+$,<br>$414.1189[M+H-C_6H_{10}O_5-H_2O]^+$,<br>$354.0969[M+H-C_8H_{14}O_7-H_2O]^+$	$472.1110[M-H-C_8H_8O]^-$,<br>$364.0829[M-H-C_{13}H_8O_4]^-$,<br>$339.0749[M-H-C_6H_{10}NO_4-C_6H_5O]^-$	HH
51	Salvianolic acid T [d]	$C_{27}H_{22}O_{12}$	10.75	539.1186<br>(0.4)	537.1049<br>(1.9)	$323.0550[M+H-C_9H_{10}O_5-H_2O]^+$,<br>$297.0757[M+H-C_9H_{10}O_5-CH_2O_2]^+$,<br>$279.0650[M+H-C_9H_{10}O_5-CO_2-H_2O]^+$,<br>$181.0495[M+H-C_{18}H_{14}O_8]^+$	$493.1155[M-H-CO_2]^-$,<br>$313.0726[M-H-CO_2-C_9H_8O_4]^-$,<br>$295.0619[M-H-CO_2-C_9H_{10}O_5]^-$	DS
52	Perlolyrine [d]	$C_{16}H_{12}N_2O_2$	10.79	265.0969<br>(–0.9)	ND	$247.0860[M+H-H_2O]^+$,<br>$206.0831[M+H-C_2H_3O_2]^+$,<br>$167.0588[M+H-C_5H_6O_2]^+$	ND	CX
53	6'-O-galloylalbiflorin [d]	$C_{30}H_{32}O_{15}$	10.89	633.1828<br>(2.0)	631.1688<br>(2.8)	$471.1382[M+H-C_6H_6O_3-2H_2O]^+$,<br>$319.1161[M+H-C_{13}H_{14}O_9]^+$,<br>$301.1060[M+H-C_{13}H_{16}O_{10}]^+$,<br>$153.0164[M+H-C_{23}H_{18}O_{11}]^+$,<br>$105.0318[M+H-C_{23}H_{28}O_{14}]^+$	$465.1416[M-H-C_7H_2O_5]^-$	CS
54	Senkyunolide S [d]	$C_{12}H_{16}O_5$	10.95	ND	239.0927<br>(1.0)	ND	$195.1025[M-H-H_2O-C_2H_2]^-$,<br>$139.0758[M-H-C_5H_8O_2]^-$,<br>$111.0812[M-H-C_6H_8O_3]^-$	CX
55	Neocarthamin [d]	$C_{21}H_{22}O_{11}$	11.01	451.1230<br>(–1.0)	449.1086<br>(–0.8)	$289.0709[M+H-C_6H_{10}O_5]^+$,<br>$169.0118[M+H-C_6H_{10}O_5-C_8H_8O]^+$	$287.0561[M-H-C_6H_{10}O_5]^-$	HH
56	Salviaflaside [d]	$C_{24}H_{26}O_{13}$	11.08	523.1441<br>(–0.9)	521.1310<br>(1.2)	$325.0903[M+H-C_9H_{10}O_5]^+$,<br>$163.0381[M+H-C_{18}H_{16}O_8]^+$	$359.0791[M-H-C_6H_{10}O_5]^-$,<br>$323.0775[M-H-C_6H_{12}O_6-H_2O]^-$,<br>$197.0462[M-H-C_{15}H_{16}O_8]^-$,<br>$161.0236[M-H-C_{18}H_{16}O_8]^-$	DS
57	Nicotiflorin [d]	$C_{27}H_{30}O_{15}$	11.42	595.1665<br>(1.3)	593.1520<br>(1.4)	$449.1078[M+H-C_6H_{10}O_4]^+$,<br>$287.0550[M+H-C_{12}H_{20}O_9]^+$	$285.0420[M-H-C_{12}H_{20}O_9]^-$	HH
58	Salvianolic acid H/I [d]	$C_{27}H_{22}O_{12}$	11.64	539.1192<br>(1.5)	537.1049<br>(1.5)	$323.0548[M+H-C_9H_{10}O_5-H_2O]^+$,<br>$295.0597[M+H-C_9H_{10}O_5-CH_2O_2]^+$,<br>$269.0803[M+H-C_{12}H_{12}O_6-H_2O]^+$,<br>$251.0696[M+H-C_{15}H_{12}O_6]^+$,<br>$181.0485[M+H-C_{18}H_{12}O_7-H_2O]^+$	$339.0521[M-H-C_9H_{10}O_5]^-$,<br>$295.0621[M-H-CO_2-C_9H_{10}O_5]^-$	DS
59	Salvianolic acid D [d]	$C_{20}H_{18}O_{10}$	11.72	419.0973	417.0829	$221.0438[M+H-C_9H_{10}O_5]^+$,	$373.0931[M-H-CO_2]^-$,	DS

				(0.1)	(0.4)	177.0538$[M+H-C_9H_8O_5-CH_2O_2]^+$,<br>159.0438$[M+H-C_{10}H_8O_6-2H_2O]^+$	197.0458$[M-H-C_{11}H_8O_5]^-$,<br>179.0354$[M-H-C_{11}H_8O_5-H_2O]^-$,<br>175.0401$[M-H-C_9H_8O_5-CH_2O_2]^-$	
60	Narcissoside [b,d]	$C_{28}H_{32}O_{16}$	11.77	625.1771<br>(1.2)	623.1624<br>(1.1)	479.1195$[M+H-C_6H_{10}O_4]^+$,<br>317.0661$[M+H-C_{12}H_{20}O_9]^+$	315.0532$[M-H-C_{12}H_{20}O_9]^-$	HH
61	Kaempferol 7-O-glucoside [d]	$C_{21}H_{20}O_{11}$	11.89	449.1083<br>(0.9)	447.0937<br>(0.9)	287.0547$[M+H-C_6H_{10}O_5]^+$	284.0331$[M-H-C_6H_{11}O_5]^-$	HH
62	Galloylpaeoniflorin [a,d]	$C_{30}H_{32}O_{15}$	12.18	633.1830<br>(2.2)	631.1682<br>(2.1)	471.1284$[M+H-C_6H_6O_3-2H_2O]^+$,<br>301.1069$[M+H-C_{13}H_{16}O_{10}]^+$,<br>153.0181$[M+H-C_{23}H_{18}O_{11}]^+$,<br>105.0331$[M+H-C_{23}H_{28}O_{14}]^+$	631.1675$[M-H]^-$	CS
63	6-Hydroxy-3-O-methyl-kaempferol-6-O-glucoside [d]	$C_{22}H_{22}O_{12}$	12.26	479.1190<br>(1.2)	477.1041<br>(0.4)	317.0654$[M+H-C_6H_8O_5]^+$	315.0506$[M-H-C_6H_{10}O_5]^-$,<br>299.0197$[M-H-C_6H_{10}O_6]^-$	HH
64	Azelaic acid [b,d]	$C_9H_{16}O_4$	12.45	189.1115<br>(–3.3)	187.0981<br>(2.9)	171.1008$[M+H-H_2O]^+$,<br>125.0941$[M+H-H_2O-CH_2O_2]^+$,<br>97.0990$[M+H-2CH_2O_2]^+$	169.0870$[M-H-H_2O]^-$,<br>125.0972$[M-H-H_2O-CO_2]^-$,<br>97.0657$[M-H-H_2O-C_3H_4O_2]^-$	DS、CS、CX、HH、JX
65	Cartormin [b,d]	$C_{27}H_{29}NO_{13}$	12.48	576.1719<br>(1.3)	574.1571<br>(0.8)	558.1623$[M+H-H_2O]^+$,<br>414.1187$[M+H-C_6H_{10}O_5]^+$,<br>306.0607$[M+H-C_6H_{10}O_5-C_7H_8O]^+$	454.0986$[M-H-C_4H_6O_3]^-$,<br>424.1034$[M-H-C_4H_6O_3-CH_2O-H2O]^-$,<br>364.0817$[M-H-C_4H_6O_3-C_7H_8O]^-$	HH
66	Salvinoside [d]	$C_{42}H_{40}O_{21}$	12.49	ND	879.2005<br>(1.8)	ND	681.1504$[M-H-C_9H_{10}O_5]^-$,<br>519.0935$[M-H-C_{18}H_{16}O_8]^-$,<br>321.0412$[M-H-C_{24}H_{26}O_{13}-2H_2O]^-$	DS
67	3',4',7-Trihydroxyflavanone [d]	$C_{15}H_{12}O_5$	12.54	273.0759<br>(0.7)	271.0615<br>(0.7)	255.0643$[M+H-H_2O]^+$,<br>163.0376$[M+H-C_6H_6O_2]^+$,<br>137.0217$[M+H-C_8H_8O_2]^+$	135.0452$[M-H-C_8H_8O_2]^-$,<br>91.0184$[M-H-C_9H_6O_3-H_2O]^-$	JX
68	Senkyunolide R [d]	$C_{12}H_{16}O_5$	12.66	ND	239.0930<br>(2.3)	ND	195.1033$[M-H-C_2H_2-H_2O]^-$,<br>154.0275$[M-H-C_5H_9O]^-$,<br>137.0230$[M-H-C_5H_{10}O_2]^-$,<br>101.0613$[M-H-C_7H_6O_3]^-$	CX
69	Tanshinone IIA-sulfonic sodium [a,d]	$C_{19}H_{17}O_6SNa$	12.87	ND	395.0543<br>(–7.1)	ND	197.0464$[M-H-C_{12}H_{14}-ONa]^-$,<br>179.0352$[M-H-C_{14}H_{15}O_2]^-$<br>161.0245$[M-H-C_{14}H_{10}O_2-Na]^-$	DS
70	Rosmarinic acid [a,b,d]	$C_{18}H_{16}O_8$	12.87	361.0919<br>(0.2)	359.0777<br>(0.9)	163.0370$[M+H-C_9H_{10}O_5]^+$,<br>145.0268$[M+H-C_9H_{10}O_5-H_2O]^+$	197.0458$[M-H-C_9H_6O_3]^-$,<br>161.0248$[M-H-C_9H_{10}O_5]^-$,<br>135.0455$[M-H-C_{10}H_8O_6]^-$	DS
71	Lithospermic acid [a,b,d]	$C_{27}H_{22}O_{12}$	13.34	539.1189<br>(0.9)	537.1047<br>(1.5)	341.0653$[M+H-C_9H_{10}O_5]^+$,<br>295.0600$[M+H-C_9H_{10}O_5-CH_2O_2]^+$,<br>279.0646$[M+H-C_9H_{10}O_5-CO_2-H_2O]^+$,<br>251.0697$[M+H-C_{15}H_{12}O_6]^+$,<br>181.0486$[M+H-C_{18}H_{14}O_8]^+$,<br>137.0224$[M+H-C_{18}H_{14}O_8-CH_2O_2]^+$	493.1147$[M-H-CO_2]^-$,<br>313.0735$[M-H-CO_2-C_9H_8O_4]^-$,<br>295.0624$[M-H-CO_2-C_9H_{10}O_5]^-$,<br>109.0300$[M-H-C_{21}H_{16}O_{10}]^-$	DS
72	Safflomin C [d]	$C_{30}H_{30}O_{14}$	13.66	615.1713<br>(0.8)	613.1577<br>(2.2)	453.1163$[M+H-C_6H_{10}O_5]^+$,<br>289.0697$[M+H-C_6H_{10}O_5-C_9H_8O_3]^+$,<br>165.0533$[M+H-C_{21}H_{22}O_{11}]^+$	551.1580$[M-H-CO_2-H_2O]^-$,<br>361.1087$[M-H-C_{12}H_8O_4-H_2O]^-$	HH
73	Safflomin C isomers [d]	$C_{30}H_{30}O_{14}$	14.07	615.1714<br>(0.9)	613.1580<br>(2.5)	453.1182$[M+H-C_6H_{10}O_5]^+$,<br>289.0704$[M+H-C_6H_{10}O_5-C_9H_8O_3]^+$,<br>165.0542$[M+H-C_{21}H_{22}O_{11}]^+$	361.1083$[M-H-C_{12}H_8O_4-H_2O]^-$	HH

74	Melitric acid B [d]	$C_{27}H_{20}O_{11}$	14.33	521.1083<br>(0.9)	519.0936<br>(0.7)	341.0664$[M+H-C_9H_8O_4]^+$,<br>323.0548$[M+H-C_9H_{10}O_5]^+$,<br>295.0603$[M+H-C_{10}H_{10}O_6]^+$,<br>181.0494$[M+H-C_{18}H_{12}O_7]^+$,<br>139.0387$[M+H-CO_2-C_{18}H_{10}O_7]^+$	339.0526$[M-H-C_9H_8O_4]^-$,<br>321.0421$[M-H-C_9H_{10}O_5]^-$,<br>295.0621$[M-H-C_{10}H_8O_6]^-$	DS
75	Salvianolic acid B [a,b,d]	$C_{36}H_{30}O_{16}$	14.34	719.1614<br>(1.0)	717.1487<br>(3.7)	521.1059$[M+H-C_9H_{10}O_5]^+$,<br>323.0545$[M+H-2C_9H_{10}O_5]^+$,<br>181.0487$[M+H-C_{27}H_{22}O_{12}]^+$	519.0928$[M-H-C_9H_{10}O_5]^-$,<br>321.0400$[M-H-2C_9H_{10}O_5]^-$,<br>295.0611$[M-H-C_9H_{10}O_5-C_{10}H_8O_6]^-$	DS
76	4-(4'-methoxyphenyl)butyric acid [d]	$C_{11}H_{14}O_3$	14.36	ND	193.0873<br>(1.3)	ND	136.0168$[M-H-C_2HO_2]^-$,<br>108.0205$[M-H-C_4H_5O_2]^-$	CS
77	Apigenin-7-O-β-D-glucuronide [b,d]	$C_{21}H_{18}O_{11}$	14.38	447.0926<br>(1.0)	ND	271.0606$[M+H-C_6H_8O_6]^+$	ND	DS
78	Salvianolic acid J [d]	$C_{27}H_{22}O_{12}$	14.43	539.1189<br>(0.8)	537.1042<br>(0.6)	323.0556$[M+H-C_9H_{10}O_5-H_2O]^+$,<br>295.0607$[M+H-C_9H_{10}O_5-CH_2O_2]^+$,<br>181.0485$[M+H-C_{18}H_{14}O_8]^+$	493.1160$[M-H-CO_2]^-$,<br>313.0726$[M-H-CO_2-C_9H_8O_4]^-$,<br>295.0626$[M-H-CO_2-C_9H_{10}O_5]^-$	DS
79	Tectorigenin [b,d]	$C_{16}H_{12}O_6$	14.92	301.0709<br>(0.6)	299.0568<br>(2.1)	269.0439$[M+H-CH_3-OH]^+$,<br>255.0644$[M+H-CHO-OH]^+$,<br>137.0219$[M+H-C_8H_4O_4]^+$	284.0336$[M-H-CH_3]^-$,<br>135.0101$[M-H-C_8H_4O_4]^-$	JX
80	Liquiritigenin [a,b,d]	$C_{15}H_{12}O_4$	15.01	257.0807<br>(–0.4)	255.0669<br>(2.5)	239.0687$[M+H-H_2O]^+$,<br>137.0220$[M+H-C_8H_8O]^+$,<br>119.0473$[M+H-C_7H_6O_3]^+$,<br>91.0527$[M+H-C_9H_{10}O_3]^+$	135.0086$[M-H-C_8H_8O]^-$,<br>119.0504$[M-H-C_7H_4O_3]^-$,<br>91.0185$[M-H-C_9H_8O_3]^-$	JX
81	Homoeriodictyol [d]	$C_{16}H_{14}O_6$	15.15	303.0866<br>(1.0)	301.0722<br>(1.5)	163.0389$[M+H-H_2O-C_7H_6O_2]^+$,<br>135.0441$[M+H-C_9H_{12}O_3]^+$,<br>107.0489$[M+H-C_{10}H_{12}O_4]^+$	191.0359$[M-H-C_6H_6O_2]^-$,<br>176.0120$[M-H-C_7H_9O_2]^-$,<br>109.0299$[M-H-C_6H_3O_2-H_2O]^-$	JX
82	Salvianolic acid A [a,b,d]	$C_{26}H_{22}O_{10}$	15.48	495.1287<br>(0.3)	493.1144<br>(0.7)	297.0757$[M+H-C_9H_{10}O_5]^+$,<br>251.0702$[M+H-C_{14}H_{12}O_4]^+$,<br>223.0756$[M+H-C_{12}H_{14}O_6-H_2O]^+$,<br>181.0491$[M+H-C_{17}H_{14}O_6]^+$	295.0615$[M-H-C_9H_{10}O_5]^-$,<br>185.0251$[M-H-C_9H_{10}O_5-C_6H_6O_2]^-$,<br>109.0301$[M-H-C_{20}H_{16}O_8]^-$	DS
83	Methyl rosmarinate [d]	$C_{19}H_{18}O_8$	15.66	375.1077<br>(0.7)	373.0931<br>(0.6)	177.0541$[M+H-C_{10}H_{14}O_4]^+$,<br>117.0331$[M+H-C_{11}H_{12}O_6-H_2O]^+$	179.0351$[M-H-C_{10}H_{10}O_4]^-$,<br>135.0451$[M-H-C_9H_7O_4-C_2H_3O_2]^-$	DS
84	(3R)-Vestitone [d]	$C_{16}H_{14}O_5$	16.08	287.0916<br>(0.8)	285.0771<br>(0.9)	163.0374$[M+H-C_7H_8O_2]^+$,<br>125.0580$[M+H-C_9H_6O_3]^+$,<br>107.0477$[M+H-C_{10}H_{12}O_3]^+$,<br>93.0321$[M+H-C_{10}H_{10}O_4]^+$	148.0154$[M-H-C_7H_5O_3]^-$,<br>135.0088$[M-H-C_9H_{10}O_2]^-$	JX
85	(3R)-Claussequinone [d]	$C_{16}H_{14}O_5$	16.35	287.0915<br>(0.4)	285.0773<br>(1.7)	163.0386$[M+H-C_7H_8O_2]^+$,<br>135.0435$[M+H-C_9H_{12}O_2]^+$,<br>123.0426$[M+H-C_9H_8O_3]^+$,<br>107.0482$[M+H-C_{10}H_{12}O_3]^+$	149.0246$[M-H-C_7H_4O_3]^-$,<br>121.0278$[M-H-C_9H_{10}O_2]^-$,<br>91.0178$[M-H-C_{10}H_{10}O_4]^-$	JX
86	2′,7-Dihydroxy-4′,5′-dimethoxyisoflavone [d]	$C_{17}H_{14}O_6$	16.57	315.0866<br>(0.9)	313.0722<br>(0.5)	283.0600$[M+H-CH_4O]^+$,<br>255.0644$[M+H-2CH_2O]^+$,<br>227.0691$[M+H-C_4H_8O_2]^+$	283.0248$[M-H-CH_2O]^-$,<br>255.0295$[M-H-2CHO]^-$,<br>227.0348$[M-H-C_4H_6O_2]^-$	JX
87	Salvianolic acid C [a,d]	$C_{26}H_{20}O_{10}$	16.78	493.1136<br>(0.5)	491.0987<br>(0.7)	295.0595$[M+H-C_9H_{10}O_5]^+$,<br>267.0640$[M+H-C_{10}H_{10}O_6]^+$,<br>239.0699$[M+H-C_{12}H_{14}O_6]^+$	311.0567$[M-H-C_9H_8O_4]^-$,<br>293.0457$[M-H-C_9H_{10}O_5]^-$,<br>265.0513$[M-H-C_{10}H_{10}O_6]^-$,<br>197.0474$[M-H-C_{17}H_{10}O_5]^-$,<br>135.0461$[M-H-C_{17}H_{10}O_5-CO_2-H_2O]^-$	DS

88	Safflospermidine A [d]	$C_{34}H_{37}N_3O_6$	16.99	584.2759<br>(0.6)	582.2610<br>(0.1)	$438.2392[M+H-C_9H_6O_2]^+$,<br>$292.2025[M+H-2C_9H_6O_2]^+$,<br>$204.1024[M+H-C_{22}H_{24}N_2O_4]^+$	$462.2003[M-H-C_8H_8O]^-$,<br>$342.1463[M-H-2C_8H_8O]^-$,<br>$119.0505[M-H-C_{26}H_{29}N_3O_5]^-$	HH
89	Methyl salvionolate A [d]	$C_{27}H_{24}O_{10}$	17.33	509.1440<br>(–0.4)	507.1301<br>(0.9)	$311.0914[M+H-C_{10}H_{12}O_4]^+$,<br>$265.0848[M+H-C_{14}H_{12}O_4]^+$,<br>$237.0926[M+H-C_{16}H_{16}O_4]^+$,<br>$181.0489[M+H-C_{17}H_{13}O_5-CH_3O]^+$	$309.0770[M-H-C_7H_6O_2-C_2H_2O_2-H_2O]^-$,<br>$197.0449[M-H-C_{17}H_{10}O_6]^-$	DS
90	(3R)-Violanone [d]	$C_{17}H_{16}O_6$	17.38	317.1018<br>(–0.5)	315.0880<br>(2.0)	$179.0696[M+H-C_7H_6O_3]^+$,<br>$163.0385[M+H-C_8H_{10}O_3]^+$,<br>$135.0434[M+H-C_{10}H_{14}O_3]^+$,<br>$107.0485[M+H-C_{11}H_{14}O_4]^+$	$285.0406[M-H-CH_2O]^-$,<br>$135.0088[M-H-C_{10}H_{12}O_3]^-$,<br>$91.0183[M-H-C_{11}H_{12}O_5]^-$	JX
91	5,3'-dihydroxy-7,4'-dimethoxyflavanone [d]	$C_{17}H_{16}O_6$	17.76	317.1017<br>(–0.9)	315.0879<br>(1.7)	$284.0680[M+H-CH_3-H_2O]^+$,<br>$148.0510[M+H-C_8H_9O_4]^+$	$250.0272[M-H-CH_2O-2OH]^-$,<br>$177.0200[M-H-C_7H_6O_3]^-$,<br>$135.0086[M-H-C_{10}H_{12}O_3]^-$	JX
92	Butein [b,d]	$C_{15}H_{12}O_5$	17.99	273.0752<br>(–1.3)	271.0619<br>(1.1)	$153.0164[M+H-C_7H_4O_2]^+$,<br>$147.0424[M+H-C_6H_4O_2-H_2O]^+$,<br>$119.0492[M+H-C_8H_{10}O_3]^+$	$151.0033[M-H-C_7H_4O_2]^-$,<br>$119.0500[M-H-C_8H_8O_3]^-$,<br>$108.0204[M-H-C_9H_7O_4]^-$	JX
93	2-Methoxy-4-(3-methoxy-1-propenyl)phenol [d]	$C_{11}H_{14}O_3$	18.02	195.1011<br>(–2.3)	193.0879<br>(3.4)	$177.0501[M+H-H_2O]^+$,<br>$123.0403[M+H-C_4H_8O]^+$	$136.0157[M-H-C_3H_5O]^-$,<br>$108.0212[M-H-C_4H_3O-H_2O]^-$	CX
94	(3R)-Vesticarpan [d]	$C_{16}H_{14}O_5$	18.15	287.0917<br>(0.9)	285.0777<br>(2.2)	$269.0811[M+H-H_2O]^+$,<br>$177.0535[M+H-C_6H_6O_2]^+$,<br>$163.0379[M+H-C_7H_8O_2]^+$,<br>$135.0428[M+H-C_9H_{12}O_2]^+$,<br>$107.0479[M+H-C_{10}H_{12}O_3]^+$	$148.0154[M-H-C_7H_5O_3]^-$,<br>$135.0088[M-H-C_9H_{10}O_2]^-$,<br>$91.0182[M-H-C_{10}H_{10}O_4]^-$	JX
95	Benzoylpaeoniflorin [a,b,d]	$C_{30}H_{32}O_{12}$	18.80	585.1974<br>(1.3)	ND	$319.1171[M+H-C_{13}H_{14}O_6]^+$,<br>$267.0860[M+H-C_{17}H_{18}O_6]^+$,<br>$249.0755[M+H-C_{17}H_{18}O_6-H_2O]^+$,<br>$197.0804[M+H-C_{13}H_{15}O_7-C_7H_5O]^+$,<br>$105.0322[M+H-C_{23}H_{28}O_{11}]^+$	ND	CS
96	Melanettin [d]	$C_{16}H_{12}O_5$	18.87	285.0761<br>(1.0)	283.0617<br>(1.8)	$252.0411[M+H-CH_3O]^+$,<br>$137.0222[M+H-C_9H_8O_2]^+$	$268.0386[M-H-CH_3]^-$,<br>$135.0086[M-H-C_9H_8O_2]^-$	JX
97	Alpinetin [b,d]	$C_{16}H_{14}O_4$	19.25	271.0967<br>(1.0)	269.0825<br>(1.6)	$167.0334[M+H-C_8H_8]^+$,<br>$152.0098[M+H-C_8H_7O]^+$,<br>$131.0484[M+H-C_7H_8O_3]^+$	$165.0199[M-H-C_8H_8]^-$,<br>$149.9948[M-H-C_8H_8O]^-$	JX
98	5'-Methoxyvestitol [d]	$C_{17}H_{18}O_5$	19.99	303.1225<br>(–0.5)	301.1088<br>(1.1)	$193.0850[M+H-C_7H_{10}O]^+$,<br>$153.0537[M+H-C_9H_{10}O_2]^+$,<br>$149.0582[M+H-C_8H_{10}O_3]^+$,<br>$125.0590[M+H-C_{10}H_{10}O_3]^+$	$271.0609[M-H-CH_2O]^-$,<br>$135.0439[M-H-C_9H_8O_2-H_2O]^-$,<br>$121.0291[M-H-C_{10}H_{12}O_3]^-$	JX
99	2'-O-Methylformononetin [d]	$C_{17}H_{14}O_5$	20.56	299.0917<br>(1.1)	297.0774<br>(1.5)	$284.0671[M+H-CH_3]^+$,<br>$252.0410[M+H-CHO-H_2O]^+$,<br>$148.0510[M+H-C_7H_5O_2-CH_2O]^+$	$282.0533[M-H-CH_3]^-$,<br>$267.0300[M-H-CH_2O]^-$,<br>$251.0342[M-H-CHO-OH]^-$,<br>$239.0346[M-H-2CHO]^-$	JX
100	Butylidenephthalide [d]	$C_{12}H_{12}O_2$	20.92	189.0908<br>(–0.8)	ND	$171.0797[M+H-H_2O]^+$,<br>$143.0848[M+H-C_2H_4-H_2O]^+$,<br>$133.0276[M+H-C_4H_8]^+$,<br>$128.0614[M+H-C_3H_7-H_2O]^+$,<br>$105.0326[M+H-C_5H_8O]^+$	ND	CX

101	(Z)-Ligustilide [d]	$C_{12}H_{14}O_2$	21.19	191.1065<br>(–0.9)	ND	149.0571[$M+H–C_3H_6]^+$,<br>135.0418[$M+H–C_4H_8]^+$,<br>105.0678[$M+H–C_5H_{10}O]^+$,<br>91.0524[$M+H–C_5H_8O–O]^+$	ND	CX
102	Formononetin [a,b,d]	$C_{16}H_{12}O_4$	21.25	269.0810<br>(0.6)	267.0670<br>(2.4)	254.0572[$M+H–CH_3]^+$,<br>237.0544[$M+H–CH_4O]^+$,<br>226.0623[$M+H–C_2H_3O]^+$	252.0431[$M–H–CH_3]^–$,<br>223.0397[$M–H–C_2H_4O]^–$	JX
103	3-O-Methylviolanone [d]	$C_{18}H_{18}O_6$	22.15	331.1184<br>(1.6)	329.1036<br>(1.8)	193.0852[$M+H–C_7H_6O_3]^+$,<br>163.0379[$M+H–C_9H_{12}O_3]^+$,<br>135.0427[$M+H–C_{11}H_{16}O_3]^+$	314.0811[$M–H–CH_3]^–$,<br>299.0577[$M–H–CH_3–CH_3O]^–$,<br>161.0256[$M–H–C_9H_{12}O_3]^–$,<br>135.0096[$M–H–C_{11}H_{14}O_3]^–$	JX
104	(3R)-Sativanone [d]	$C_{17}H_{16}O_5$	22.64	301.1074<br>(0.9)	299.0931<br>(2.1)	283.0959[$M+H–H_2O]^+$,<br>163.0383[$M+H–C_7H_6O_3]^+$,<br>135.0424[$M+H–C_{10}H_{14}O_2]^+$,<br>107.0474[$M+H–C_{11}H_{14}O_3]^+$	284.0696[$M–H–CH_3]^–$,<br>269.0463[$M–H–CH_2O]^–$,<br>135.0086[$M–H–C_{10}H_{12}O_2]^–$,<br>91.0188[$M–H–C_{11}H_{12}O_4]^–$	JX
105	Senkyunolide E [d]	$C_{12}H_{12}O_3$	22.98	205.0855<br>(–2.2)	203.0724<br>(5.0)	187.0746[$M+H–H_2O]^+$,<br>144.0549[$M+H–C_3H_9O]^+$,<br>131.0839[$M+H–C_4H_{10}O]^+$,<br>107.0478[$M+H–C_5H_6O_2]^+$	173.0245[$M–H–C_2H_6]^–$,<br>160.0165[$M–H–C_2H_2–H_2O]^–$,<br>132.0212[$M–H–C_4H_7O]^–$	CX
106	Pinocembrin [b,d]	$C_{15}H_{12}O_4$	23.21	257.0806<br>(–0.8)	255.0673<br>(3.8)	239.0687[$M+H–H_2O]^+$,<br>153.0175[$M+H–C_8H_8O]^+$,<br>131.0476[$M+H–C_6H_6O_3]^+$,<br>103.0526[$M+H–C_7H_6O_4]^+$	213.0556[$M–H–C_2H_2O]^–$,<br>151.0034[$M–H–C_8H_8]^–$	JX
107	(E)-Ligustilide [d]	$C_{12}H_{14}O_2$	24.04	191.1062<br>(–2.2)	ND	145.0987[$M+H–C_3H_{10}]^+$,<br>115.0526[$M+H–C_6H_4]^+$,<br>105.0677[$M+H–C_5H_{10}O]^+$,<br>91.0526[$M+H–C_5H_8O–O]^+$	ND	CX

Note: [a] Confirmation in comparison with authentic standards. [b] Confirmation in comparison with mass spectral library (Natural Products HR-MS/MS Spectral Library, Version 1.0; AB Sciex, Foster City, CA, USA). [c] HILIC-MS. [d] RPLC-MS. ND=Not detect. DS: Danshen (*Salviae Miltiorrhizae Radix Et Rhizoma*). CS: Chishao (*Paeoniae Radix Rubra*). CX: Chuanxiong (*Chuanxiong Rhizoma*). HH: Honghua (*Carthami Flos*). JX: Jiangxiang (*Dalbergiae Odoriferae Lignum*).

Table S2. Potentially 39 active ingredients of JZGX.

No.	Compounds
A	Malic acid
B	Protocatechuic acid
C	Protocatechualdehyde
D	Caffeic acid
E	Azelaic acid
DS1	Danshensu
DS2	Tanshinone IIA-sulfonic sodium
DS3	Methyl rosmarinate
CS1	Gallic acid
CS2	Methyl gallate
CX1	Phthalic acid
CX2	Vanillin
CX3	Ferulic acid
CX4	4-(4'-methoxyphenyl)butyric acid
CX5	Butylidenephthalide
CX6	(Z)-Ligustilide
CX7	Senkyunolide E
CX8	(E)-Ligustilide
HH1	1,2,3,4-tetrahydro-3-carboxy-2-carboline
HH2	*p*-Coumaric acid
JX1	3',4',7-Trihydroxyflavanone
JX2	Tectorigenin

JX3	Liquiritigenin
JX4	Homoeriodictyol
JX5	(3R)-Vestitone
JX6	(3R)-Claussequinone
JX7	2',7-Dihydroxy-4',5'-dimethoxyisoflavone
JX8	(3R)-Violanone
JX9	5,3'-dihydroxy-7,4'-dimethoxyflavanone
JX10	Butein
JX11	(3R)-Vesticarpan
JX12	Melanettin
JX13	Alpinetin
JX14	5'-Methoxyvestitol
JX15	2'-O-Methylformononetin
JX16	Formononetin
JX17	3-O-Methylviolanone
JX18	(3R)-Sativanone
JX19	Pinocembrin

Note: DS: Danshen (*Salviae Miltiorrhizae Radix Et Rhizoma*). CS: Chishao (*Paeoniae Radix Rubra*). CX: Chuanxiong (*Chuanxiong Rhizoma*). HH: Honghua (*Carthami Flos*). JX: Jiangxiang (*Dalbergiae Odoriferae Lignum*).

Table S3. The 37 Key targets of JZGX in treating cardiovascular diseases.

No.	Gene symbol	Protein name	Degree	Betweenness centrality	Closeness centrality
1	ALB	Albumin	236	0.1607	0.6707
2	EGFR	Epidermal growth factor receptor	180	0.0593	0.6049
3	SRC	Proto-oncogene tyrosine-protein kinase Src	162	0.0329	0.5920
4	PTGS2	Prostaglandin G/H synthase 2	160	0.0513	0.5827
5	ESR1	Estrogen receptor	156	0.0649	0.5842
6	STAT3	Signal transducer and activator of transcription 3	154	0.0324	0.5812
7	PPARG	Peroxisome proliferator-activated receptor gamma	154	0.0737	0.5812
8	HSP90AA1	Heat shock protein HSP 90-alpha	146	0.0357	0.5707
9	CCND1	G1/S-specific cyclin-D1	132	0.0242	0.5606
10	MMP9	Matrix metalloproteinase-9	126	0.0232	0.5509
11	TLR4	Toll-like receptor 4	116	0.0157	0.5324
12	MAPK1	Mitogen-activated protein kinase 1	112	0.0217	0.5415
13	PPARA	Peroxisome proliferator-activated receptor alpha	100	0.0217	0.5349
14	AR	Androgen receptor	94	0.0195	0.5298
15	IL2	Interleukin-2	94	0.0106	0.5311
16	MMP2	72 kDa type IV collagenase	92	0.0053	0.5236
17	ACE	Angiotensin-converting enzyme	90	0.0213	0.5175
18	JAK2	Tyrosine-protein kinase JAK2	88	0.0083	0.5175
19	HDAC1	Histone deacetylase 1	88	0.0173	0.5211
20	ICAM1	Intercellular adhesion molecule 1	88	0.0043	0.5103
21	CYP3A4	Cytochrome P450 3A	86	0.0206	0.5224
22	PIK3CA	Phosphatidylinositol 4,5-bisphosphate 3-kinase catalytic subunit alpha isoform	86	0.0060	0.4933

23	KDR	Vascular endothelial growth factor receptor 2	82	0.0050	0.5115
24	HSP90AB1	Heat shock protein HSP 90-beta	82	0.0060	0.5211
25	CDK4	Cyclin-dependent kinase 4	82	0.0142	0.5068
26	RELA	GTP pyrophosphokinase	80	0.0073	0.5163
27	MAPK8	Mitogen-activated protein kinase 8	78	0.0046	0.5211
28	MCL1	Induced myeloid leukemia cell differentiation protein Mcl-1	78	0.0055	0.5057
29	SERPINE1	SERPINE1 mRNA-binding protein 1	78	0.0044	0.5045
30	PTPRC	Receptor-type tyrosine-protein phosphatase C	76	0.0052	0.5045
31	PARP1	Poly [ADP-ribose] polymerase 1	76	0.0125	0.4966
32	MPO	Myeloperoxidase	74	0.0060	0.4922
33	GSK3B	Glycogen synthase kinase-3 beta	74	0.0049	0.5103
34	CDK1	Cyclin-dependent kinase 1	72	0.0091	0.4978
35	REN	Renin	72	0.0127	0.4944
36	CDK2	Cyclin-dependent kinase 2	70	0.0052	0.4978
37	CCNB1	G2/mitotic-specific cyclin-B1	70	0.0044	0.4978

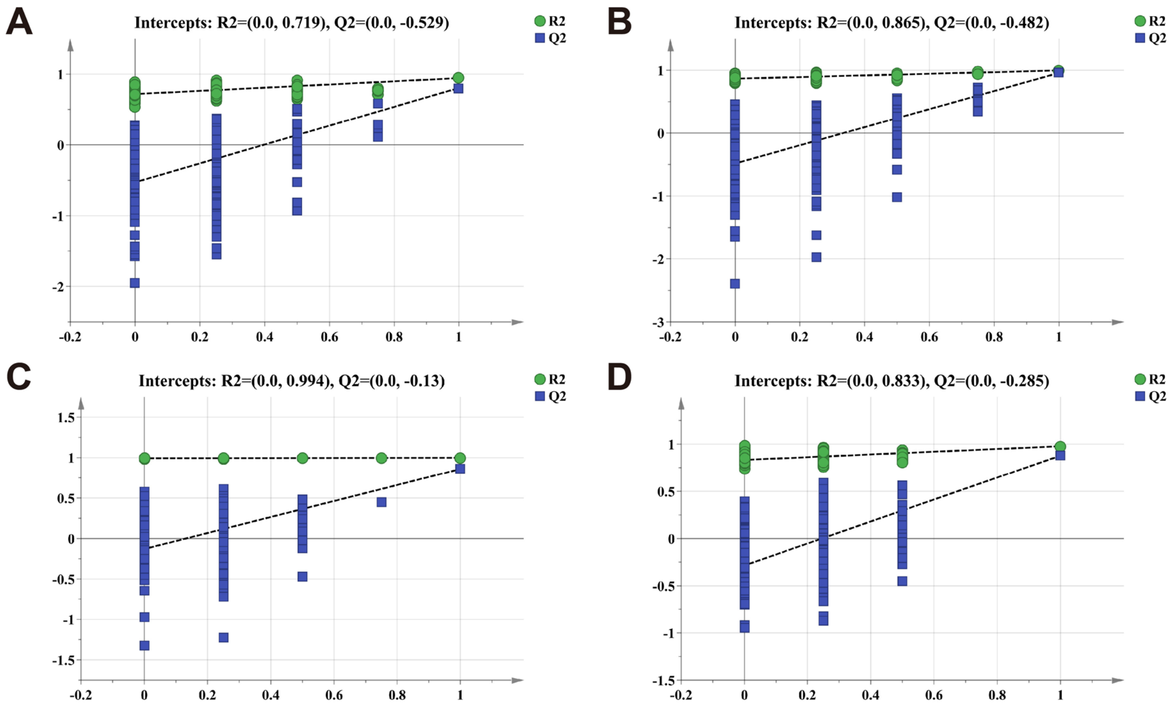

Figure S1. The permutation test on the OPLS-DA of the CON and MOD groups in the negative **(A)** and positive **(B)** modes, the MOD and JZGX groups in the negative **(C)** and positive **(D)** mode.

Table S4. The 107 differential metabolites significantly regulated by JZGX in serum metabolomic analysis.

No.	Retention Time (min)	Measured mass (Da)	Molecular Formula	Metabolites	VIP		FC		Trend	
					MOD vs. CON	JZGX vs. MOD	MOD vs. CON	JZGX vs. MOD	MOD vs. CON	JZGX vs. MOD
1	1.11	217.0702	C10H17NO4	Propenoylcarnitine	1.50	1.94	0.42	7.62	$\downarrow^{**}$	$\uparrow^{\#\#}$
2	1.14	286.0599	C15H29NO4	Octanoylcarnitine	1.39	1.29	0.51	2.25	$\downarrow^{**}$	$\uparrow^{\#\#}$
3	1.58	245.1558	C12H21NO4	Tiglylcarnitine	1.33	1.45	0.52	1.91	$\downarrow^{**}$	$\uparrow^{\#\#}$
4	1.72	302.0331	C14H25NO6	Pimelylcarnitine	1.22	1.06	0.45	1.40	$\downarrow^{**}$	$\uparrow^{\#}$
5	2.86	339.1522	C20H32O3	8-HETE	1.04	1.51	0.68	2.50	$\downarrow^{*}$	$\uparrow^{\#\#}$
6	2.88	178.9205	C4H6O5	Malic acid	1.12	1.52	0.79	1.47	$\downarrow^{**}$	$\uparrow^{\#\#}$
7	2.89	247.1281	C12H23NO4	Isovalerylcarnitine	1.08	1.72	0.82	1.89	$\downarrow^{*}$	$\uparrow^{\#\#}$
8	3.08	188.9493	C11H14N2O	5-Methoxytryptamine	1.39	1.33	0.66	1.24	$\downarrow^{**}$	$\uparrow^{\#\#}$
9	4.96	144.9564	C5H10N2O3	Glutamine	1.53	1.02	0.54	1.21	$\downarrow^{**}$	$\uparrow^{\#}$
10	5.05	845.7911	C48H94NO8P	PC(40:1)	1.47	1.34	0.27	3.50	$\downarrow^{**}$	$\uparrow^{\#}$
11	5.05	930.2719	C49H83O13P	PI(18:0/22:6(4Z,7Z,10Z,13Z,16Z,19Z))	1.48	1.23	0.27	3.38	$\downarrow^{**}$	$\uparrow^{\#}$
12	5.20	460.2382	C25H47NO5	3-Hydroxy-11Z-octadecenoylcarnitine	1.05	1.20	0.61	4.15	$\downarrow^{*}$	$\uparrow^{\#}$
13	5.80	467.3084	C24H40O5	Cholic acid	1.46	1.56	10.17	0.42	$\uparrow^{**}$	$\downarrow^{\#\#}$
14	5.82	465.3037	C27H46O4S	Cholesterol sulfate	1.45	1.49	10.03	0.45	$\uparrow^{**}$	$\downarrow^{\#\#}$
15	6.13	460.9153	C26H38O7	Retinyl beta-glucuronide	1.50	1.67	2.45	0.65	$\uparrow^{**}$	$\downarrow^{\#\#}$
16	6.22	857.5176	C55H102O6	TG(18:1(11Z)/16:0/18:1(11Z))	1.32	1.45	2.23	0.72	$\uparrow^{**}$	$\downarrow^{\#\#}$
17	6.26	352.8555	C20H34O5	11,12,15-THETA	1.47	1.53	0.33	1.43	$\downarrow^{**}$	$\uparrow^{\#\#}$
18	6.68	355.8236	C24H41NO2	Adrenoyl ethanolamide	1.60	1.74	0.07	1.97	$\downarrow^{**}$	$\uparrow^{\#\#}$
19	7.11	886.5546	C30H48N7O16P3S	3-trans,5-cis-Octadienoyl-CoA	1.37	1.73	3.32	0.47	$\uparrow^{**}$	$\downarrow^{\#\#}$
20	7.15	775.6161	C44H88NO7P	PC(18:0/P-18:0)	1.34	1.63	0.44	4.39	$\downarrow^{**}$	$\uparrow^{\#\#}$
21	7.22	855.5012	C55H100O6	TG(16:0_36:3)	1.34	1.39	5.77	0.46	$\uparrow^{**}$	$\downarrow^{\#\#}$

22	7.22	861.5519	C45H83O13P	PI(18:1(9Z)/18:1(11Z))	1.51	1.40	4.37	0.74	↑**	↓##
23	7.34	423.3313	C15H23N6O5S	S-Adenosylmethionine	1.50	1.76	0.31	1.96	↓**	↑##
24	7.40	298.6263	C18H32O2	Linoleic acid	1.57	1.48	4.11	0.59	↑**	↓##
25	7.43	773.6263	C44H86NO7P	PC(18:0/P-18:1(11Z))	1.25	1.75	0.59	3.29	↓**	↑##
26	7.53	596.3552	C28H58NO7P	LysoPC(20:0/0:0)	1.31	1.37	1.85	0.75	↑**	↓##
27	7.56	654.9120	C41H80O5	DG(18:0/20:0)	1.52	1.37	3.48	0.57	↑**	↓#
28	7.60	653.4029	C42H85NO3	Cer(d18:0/24:0)	1.51	1.75	4.14	0.26	↑**	↓##
29	7.79	593.2716	C37H70O5	DG(16:0/18:1)	1.32	1.06	2.99	0.73	↑**	↓#
30	7.79	311.6339	C18H30O4	12(13)Ep-9-KODE	1.39	1.41	3.61	0.40	↑**	↓#
31	7.81	831.9713	C48H96NO7P	PC(O-40:1)	1.39	1.31	2.39	0.56	↑**	↓#
32	7.83	806.9637	C51H96O6	TG(16:0_32:1)	1.30	1.46	2.76	0.31	↑*	↓##
33	7.86	819.4669	C48H84NO7P	PC(40:2)	1.28	1.63	2.24	0.28	↑**	↓##
34	7.90	807.4844	C42H79NO13	LacCer(d18:1/12:0)	1.37	1.50	2.46	0.36	↑**	↓##
35	7.96	783.9698	C44H80NO8P	PC(36:4)	1.63	1.30	5.48	0.64	↑**	↓#
36	8.01	887.5524	C47H85O13P	PI(16:0/22:3(10Z,13Z,16Z))	1.43	1.34	3.66	0.75	↑**	↓##
37	8.02	426.3557	C25H46NO4	Linoleyl carnitine	1.40	1.44	3.52	0.40	↑**	↓##
38	8.09	415.3599	C23H43NO5	3-Hydroxy-9-hexadecenoylcarnitine	1.50	1.32	4.63	0.52	↑**	↓#
39	8.26	788.9990	C45H91N2O6P	SM(d18:1/22:0)	1.38	1.57	2.73	0.28	↓**	↑##
40	8.26	789.4982	C44H86NO8P	PC(36:1)	1.32	1.68	2.26	0.23	↓**	↑##
41	8.31	534.3520	C26H46NO7P	LysoPC(18:4(6Z,9Z,12Z,15Z)/0:0)	1.55	1.27	3.31	0.64	↑**	↓#
42	8.37	562.3115	C36H69NO3	Cer(d18:1/18:1(9Z))	1.21	1.30	2.25	0.51	↑**	↓##
43	8.47	560.3127	C28H48NO7P	LysoPC(20:5(5Z,8Z,11Z,14Z,17Z)/0:0)	1.19	1.45	1.65	0.45	↑*	↓##
44	8.65	546.3567	C10H16N5O14P3	Guanosine triphosphate	1.57	1.37	3.07	0.57	↑**	↓#
45	8.68	709.4994	C41H78O5	DG(14:0/24:1(15Z)/0:0)	1.54	1.58	18.46	0.55	↑**	↓##
46	8.75	512.3598	C24H48NO7P	LysoPC(16:1/0:0)	1.33	1.51	1.54	0.56	↑**	↓##

47	8.76	274.6520	C12H19NO6	Glutaconylcarnitine	1.52	1.26	1.79	0.68	↑**	↓#
48	8.76	538.3129	C26H50NO7P	LysoPC(18:2/0:0)	1.16	1.36	1.34	0.65	↑*	↓##
49	8.77	659.5117	C39H66O5	DG(14:0/22:5(4Z,7Z,10Z,13Z,16Z)/0:0)	1.57	1.44	35.35	0.61	↑**	↓##
50	8.77	560.3720	C33H60O5	DG(14:1(9Z)/16:1(9Z)/0:0)	1.78	1.49	14.76	0.65	↑**	↓##
51	8.78	284.1429	C18H34O2	Oleic acid	1.14	1.23	1.48	0.64	↑*	↓#
52	8.81	721.5473	C37H77N2O6P	SM(d18:0/14:0)	1.53	1.51	19.21	0.60	↑**	↓##
53	8.97	647.3222	C32H66NO7P	LysoPC(24:0/0:0)	1.56	1.49	3.90	0.53	↑**	↓##
54	8.99	523.3593	C26H52NO7P	LysoPC(18:1/0:0)	1.67	1.52	3.36	0.63	↑**	↓##
55	9.11	648.3482	C42H83NO3	Cer(d18:1/24:0)	1.32	1.18	2.06	0.77	↑**	↓#
56	9.14	605.3072	C36H71NO3	Cer(d18:1/18:0)	1.46	1.48	2.27	0.53	↑**	↓##
57	9.21	309.9010	C17H29NO4	2-trans,4-cis-Decadienoylcarnitine	1.33	1.63	6.69	0.31	↑**	↓##
58	9.23	570.3525	C32H65NO3	Cer(d18:0/14:0)	1.18	1.18	1.53	0.76	↑**	↓#
59	9.26	231.8862	C8H11NO5S	Dopamine 3-O-sulfate	1.45	1.68	8.78	0.33	↑**	↓##
60	9.31	383.9412	C20H37NO3	Oleoyl glycine	1.20	1.49	13.37	0.22	↑**	↓##
61	9.31	363.2841	C22H35NO3	N-Arachidonoylglycine	1.65	1.37	7.22	0.58	↑**	↓#
62	9.4	578.2395	C30H62NO7P	LysoPC(22:0/0:0)	1.47	1.57	0.32	1.54	↓**	↑##
63	9.42	599.3021	C30H58NO7P	LysoPC(22:2(13Z,16Z)/0:0)	1.53	1.35	3.74	0.48	↑**	↓##
64	9.51	885.5485	C47H83O13P	PI(16:0/22:4(10Z,13Z,16Z,19Z))	1.19	1.59	2.75	0.41	↑*	↓##
65	9.77	624.3199	C39H74O5	DG(14:0/22:1(13Z)/0:0)	1.68	1.21	9.04	0.65	↑**	↓#
66	9.83	522.3542	C9H16N3O14P3	Cytidine triphosphate	1.68	1.50	3.37	0.66	↑**	↓##
67	9.84	724.3376	C42H79NO8	GlcCer(d18:1/18:1)	1.20	1.24	1.57	0.70	↑**	↓##
68	9.97	307.1586	C9H17NO8	Neuraminic acid	1.41	1.52	12.87	0.06	↑**	↓##
69	10.31	735.5248	C51H92O2	CE(24:0)	1.38	1.39	2.74	0.66	↑**	↓##
70	10.56	813.5464	C47H95N2O6P	SM(d18:1/24:0)	1.35	1.22	2.71	0.75	↑**	↓#
71	10.58	314.8792	C17H33NO4	Decanoylcarnitine	1.25	1.14	0.14	1.30	↓**	↑#

72	10.72	564.3969	C33H64O5	DG(14:0/16:0/0:0)	1.69	1.23	2.00	0.78	↑**	↓#
73	10.72	627.5353	C30H47N3O9S	Leukotriene C4	1.27	1.47	2.84	0.24	↑*	↓##
74	10.75	373.2698	C13H18N4O9	Succinylaminoimidazole carboxamide riboside	1.04	1.45	2.55	0.39	↑*	↓##
75	10.75	591.6623	C37H68O5	DG(16:0/18:2)	1.08	1.54	2.11	0.40	↑*	↓##
76	10.80	403.2359	C27H44O	7-Dehydrocholesterol	1.66	1.22	5.46	0.61	↑**	↓#
77	11.26	248.8853	C11H12N2O2	L-Tryptophan	1.48	1.06	0.08	1.80	↓**	↑#
78	11.43	326.1884	C20H41NO2	Stearoylethanolamide	1.41	1.65	0.48	1.65	↓**	↑##
79	11.43	455.1025	C23H42NO4	9,12-Hexadecadienoylcarnitine	1.14	1.29	0.32	1.64	↓*	↑#
80	11.45	578.4177	C33H62O5	DG(14:0/16:1(9Z)/0:0)	1.53	1.24	1.83	0.75	↑**	↓##
81	11.53	238.8578	C6H12N2O4S2	L-Cystine	1.15	1.27	0.02	2.35	↓*	↑##
82	11.64	645.4852	C40H79NO3	Cer(d18:1/22:0)	1.78	1.37	3.80	0.69	↑**	↓##
83	12.05	508.4115	C10H16N5O13P3	Adenosine triphosphate	1.57	1.41	2.22	0.67	↑**	↓##
84	12.14	340.2055	C19H35NO4	trans-2-Dodecenoylcarnitine	1.21	1.11	0.61	1.66	↓**	↑#
85	12.22	844.5682	C50H90NO8P	PC(42:5)	1.08	1.06	1.89	0.69	↑*	↓#
86	12.55	509.2375	C26H54NO6P	LysoPC(P-18:0/0:0)	1.37	1.42	2.31	0.57	↑**	↓#
87	12.84	673.5348	C37H71O8P	PA(16:0/18:1(11Z))	1.49	1.39	14.34	0.61	↑**	↓##
88	13.27	592.4323	C37H66O5	DG(14:0/20:3(5Z,8Z,11Z)/0:0)	1.65	1.33	2.35	0.66	↑**	↓#
89	13.79	689.5581	C38H74NO7P	PC(O-30:2)	1.35	1.45	3.42	0.38	↑**	↓##
90	14.05	265.2170	C13H14N2O3	N-Acetyltryptophan	1.55	1.13	3.38	0.68	↑**	↓#
91	14.08	759.5659	C43H89N2O6P	SM(d18:0/20:0)	1.46	1.30	22.75	0.54	↑**	↓##
92	14.24	606.4500	C35H66O5	DG(14:0/18:1)	1.33	1.36	1.58	0.62	↑**	↓##
93	14.27	881.5167	C57H102O6	TG(16:0_38:4)	1.26	1.32	3.27	0.63	↑**	↓##
94	14.44	674.5294	C37H69O8P	PA(16:0/18:2(9Z,12Z))	1.77	1.76	8.42	0.43	↑**	↓##
95	14.77	673.5270	C34H68NO7P	LysoPC(26:1/0:0)	1.75	1.78	8.70	0.51	↑**	↓##

96	14.91	607.4509	C32H64NO7P	LysoPC(24:1(15Z)/0:0)	1.25	1.38	1.57	0.63	↑**	↓##
97	15.48	884.5359	C48H90NO8P	PC(40:3)	1.31	1.42	2.33	0.59	↑**	↓##
98	15.49	700.5456	C47H80O2	CE(20:2(6Z,9Z))	1.36	1.30	2.60	0.56	↑**	↓#
99	15.72	811.6051	C46H84NO8P	PC(38:4)	1.06	1.48	1.97	0.31	↑*	↓##
100	15.92	750.5382	C38H76NO8P	PC(30:0)	1.09	1.04	1.83	0.73	↑*	↓#
101	16.14	747.5635	C40H77O10P	PG(16:0/18:1(11Z))	1.32	1.30	3.73	0.65	↑**	↓##
102	16.45	778.5745	C44H78NO8P	PC(36:5)	1.07	1.29	1.70	0.63	↑*	↓##
103	16.48	800.6095	C44H79O10P	PG(16:0/22:4(7Z,10Z,13Z,16Z))	1.22	1.41	1.95	0.46	↑*	↓##
104	18.32	112.9857	C4H4O5	Oxalacetic acid	1.22	1.37	0.50	1.43	↓**	↑##
105	18.65	609.3171	C35H64O5	DG(14:0/18:2)	1.33	1.48	7.31	0.19	↑**	↓##
106	19.25	477.3604	C27H44O4	3beta,7alpha-Dihydroxy-5-cholestenoate	1.25	1.47	3.29	0.41	↑**	↓##
107	19.29	445.3668	C24H30O8	Estrone glucuronide	1.22	1.44	3.40	0.42	↑**	↓##

Note: $^{*}p < 0.05$ vs. CON group, $^{**}p < 0.01$ vs. CON group, $^{\#}p < 0.05$ vs. MOD group, $^{\#\#}p < 0.01$ vs. MOD group.

Table S5. Molecular docking of 39 active ingredients and PPARs.

No.	PPARA	PPARG
A	-5.1	-5.0
B	-5.8	-5.8
C	-5.3	-5.3
D	-6.4	-6.5
E	-5.0	-5.4
DS1	-6.1	-6.4
DS2	-9.0	-9.6
DS3	-8.4	-8.6
CS1	-6.0	-6.1
CS2	-5.6	-6.1
CX1	-6.3	-6.1
CX2	-5.5	-5.4
CX3	-6.2	-6.4
CX4	-5.9	-6.1
CX5	-6.4	-7.2
CX6	-7.1	-6.9
CX7	-6.7	-7.2
CX8	-6.7	-7.6
HH1	-7.6	-7.7
HH2	-6.2	-6.1
JX1	-8.4	-9.0
JX2	-8.0	-8.1
JX3	-7.8	-8.2
JX4	-7.7	-8.4
JX5	-8.3	-8.6
JX6	-8.0	-8.1
JX7	-7.3	-8.4
JX8	-6.1	-8.1
JX9	-8.4	-8.8
JX10	-7.7	-8.1
JX11	-8.4	-9.0
JX12	-8.7	-8.5
JX13	-6.8	-8.1
JX14	-8.1	-8.5
JX15	-7.6	-8.3
JX16	-8.3	-8.9
JX17	-7.4	-8.3
JX18	-7.9	-8.1
JX19	-7.7	-8.0

Table S6. The general information of reference standards.

No.	Coumpounds	Purity (%)	Batch number	Source
1	D-Mannitol	99.6	140651-201403	National Institute for Control of Biological and Pharmaceutical Products of China (Beijing, China)
2	L-Arginine	100.0	140685-201305	
3	Sodium Danshensu	98.1	110855-201614	
4	Protocatechualdehyde	99.3	110810-201608	
5	Chlorogenic acid	96.2	110753-201415	
6	Hydroxysafflor yellow A	93.1	111637-201810	
7	Ferulic acid	99.0	110773-201614	
8	Rutin	92.6	100080-201409	
9	Rosmarinic acid	90.5	111871-201706	
10	Salvianolic acid B	94.1	111562-201716	
11	Raffinose	99.0	200902	Chengnuo Biotech Co., Ltd. (Zhongshan, China)
12	D-Stachyose	99.2	19083009	
13	Malic acid	98.0	181103	
14	Adenosine	98.0	18041902	
15	Gallic acid	98.0	18032703	
16	Protocatechuic acid	98.0	18082901	
17	Oxypaeoniflorin	98.0	19053107	
18	Caffeic acid	98.0	17122804	
19	Albiflorin	98.0	39011-90-0	
20	Vanillin	98.0	17062111	
21	*p*-Coumaric acid	98.0	18011605	
22	Paeoniflorin	98.0	23180-57-6	
23	Galloylpaeoniflorin	98.0	CN90831	
24	Lithospermic acid	98.0	200511	
25	Liquiritigenin	98.0	200427	
26	Salvianolic acid A	98.7	20061605	
27	Salvianolic acid C	98.0	18030102	
28	Benzoylpaeoniflorin	98.1	20032703	
29	Formononetin	98.0	200902	
30	D-Glucose	98.0	S10S9I69833	Yuanye Biotech Co., Ltd. (Shanghai, China)
31	Sucrose	98.0	H22J9R53193	
32	Citric acid	98.0	SM0426GA14	
33	L-Phenylalanine	98.0	H20N8H48638	
34	Tanshinone IIA-sulfonic sodium	98.0	Z15J9B63708	
